# Schooling substantially improves intelligence, but neither lessens nor widens the impacts of socioeconomics and genetics

**DOI:** 10.1038/s41539-022-00148-5

**Published:** 2022-12-15

**Authors:** Nicholas Judd, Bruno Sauce, Torkel Klingberg

**Affiliations:** 1 Department of Neuroscience, Karolinska Institute, Stockholm, Sweden; 2 Cognitive Neuroscience Department, Donders Institute for Brain, Cognition, and Behavior, Radboud University Medical Center, Nijmegen, The Netherlands; 3 Department of Biological Psychology, Vrije Universiteit Amsterdam, Amsterdam, The Netherlands

**Keywords:** Human behaviour, Intelligence

## Abstract

Schooling, socioeconomic status (SES), and genetics all impact intelligence. However, it is unclear to what extent their contributions are unique and if they interact. Here we used a multi-trait polygenic score for cognition (cogPGS) with a quasi-experimental regression discontinuity design to isolate how months of schooling relate to intelligence in 6567 children (aged 9–11). We found large, independent effects of schooling (*β* ~ 0.15), cogPGS (*β* ~ 0.10), and SES (*β* ~ 0.20) on working memory, crystallized (*c*IQ), and fluid intelligence (*f*IQ). Notably, two years of schooling had a larger effect on intelligence than the lifetime consequences, since birth, of SES or cogPGS-based inequalities. However, schooling showed no interaction with cogPGS or SES for the three intelligence domains tested. While schooling had strong main effects on intelligence, it did not lessen, nor widen the impact of these preexisting SES or genetic factors.

## Introduction

Inter-individual differences in intelligence predict a variety of important life outcomes, such as life satisfaction, mortality, and educational achievement (EA)^1–3^, showing correlations higher than 0.7 with achievement^4,5^. There has been considerable controversy on the source of these differences. Decades of research have shown intelligence to be highly associated with genetic differences (heritability estimates around 0.6) but also impacted by experiences and context (called here “environmental factors”)^6–12^.

Schooling is an important environmental factor, having a large impact on intelligence in children^13–15^. This has been shown in longitudinal studies controlling for prior intelligence^16^ and in studies evaluating the cognitive effects of policy changes regarding compulsory schooling^17^. A third method to evaluate the impact of schooling (called regression discontinuity designs) exploits the fact children are put in a grade based on an arbitrary age cut-off, and allows us to separate the effect of chronological age from months of schooling^18^. Studies using this method also replicate the findings that schooling affects intelligence^19,20^. Thus, many lines of research provide converging evidence that schooling can change abilities often thought to be “fixed”, such as fluid intelligence and working memory, with estimates of one year of additional schooling benefitting cognitive abilities somewhere between 1 to 5 IQ points, or 0.07 to 0.3 SD^13,19,21^. What is less clear is how the impact of schooling *interacts* with the environments that children experience and with their genetic predispositions—in other words, do school settings amplify preexisting differences (i.e., “rich-gets-richer”), or conversely, weaken existing differences between children (i.e., “catch up”).

Much of a child’s environment is captured by socioeconomic status (SES), a summary measure usually comprised of household income, parental education, and neighborhood quality^22^. While commonly implicated in initial differences in intelligence, SES has also been found to widen existing differences throughout development^23–25^. Recently, research on SES has been criticized for neglecting the role of genetics—as parents not only hand down environments but also genes^26,27^. For example, a genetically informed twin study found a strong genetic influence on SES and its association with intelligence^28^. While there is a large body of research looking at how preexisting SES differences interplay with years of schooling^29^, none of these previous studies have incorporated genetics.

Fortunately, genome-wide association studies (GWAS) with extremely large sample sizes and the viability of polygenic scores, have made it possible to incorporate genetically informative measures into a study. A polygenic score is an index that combines thousands/millions of DNA regions (each with only a tiny effect on the trait of interest) and gives a value to each individual representing their genetic propensity. Relevant to us here, a multi-trait cognitive polygenic score (cogPGS) was recently shown to predict 7–10% of the variance in cognitive performance^30^. This and similar polygenic scores correlate moderately (r ~0.3) with SES^31,32^, but little is known about their unique contributions to different domains of intelligence. And even less known if, or how, they interplay with schooling.

Gene-by-environment (GE) interplay is often proposed as an explanation of how intelligence can show high heritability alongside malleability^33–36^. To date, there are only a few studies on GE interplay using polygenic scores. Of these studies, most have focused on SES as the environmental variable of interest and none have used the environmental variable of schooling, which is particularly relevant to intelligence. Unfortunately, GE-interplay results with SES have been inconsistent for educational achievement/attainment—some positive, negative, or null^37–41^. While there is a high correlation between educational achievement and intelligence, educational achievement is a broader concept thought to also include personality characteristics such as consciousness and openness to experience^42,43^. Furthermore, we are not aware of any research examining if schooling moderates’ genetic effects on intelligence.

Schooling, SES, and genetics thus represent three substantial contributors to intelligence, but it is unclear to what extent their contributions are (1) unique and (2) interact with one another. In a sample of 3rd to 5th grade children, we estimated the unique contributions of a year of schooling, SES, and cogPGS on crystallized intelligence (*c*IQ), fluid intelligence (*f*IQ), and working memory (WM). We chose these domains of intelligence since they are particularly important for educational outcomes^4,16,44^. WM was included since WM has shown to be heavily impacted by schooling^21^, malleable^45^, and potentially relevant for GE-interplay^46^. Our main aim was to examine if GE-interplay is present for these domains of cognition. Specifically, we tested if the effect of (1) schooling or (2) SES is moderated by cogPGS, and (3) a three-way interaction between schooling, SES, and cogPGS. These interactions allowed us to test if schooling enhances or compensates for preexisting genetic and environmental inequity.

## Results

**Fig. 1:**
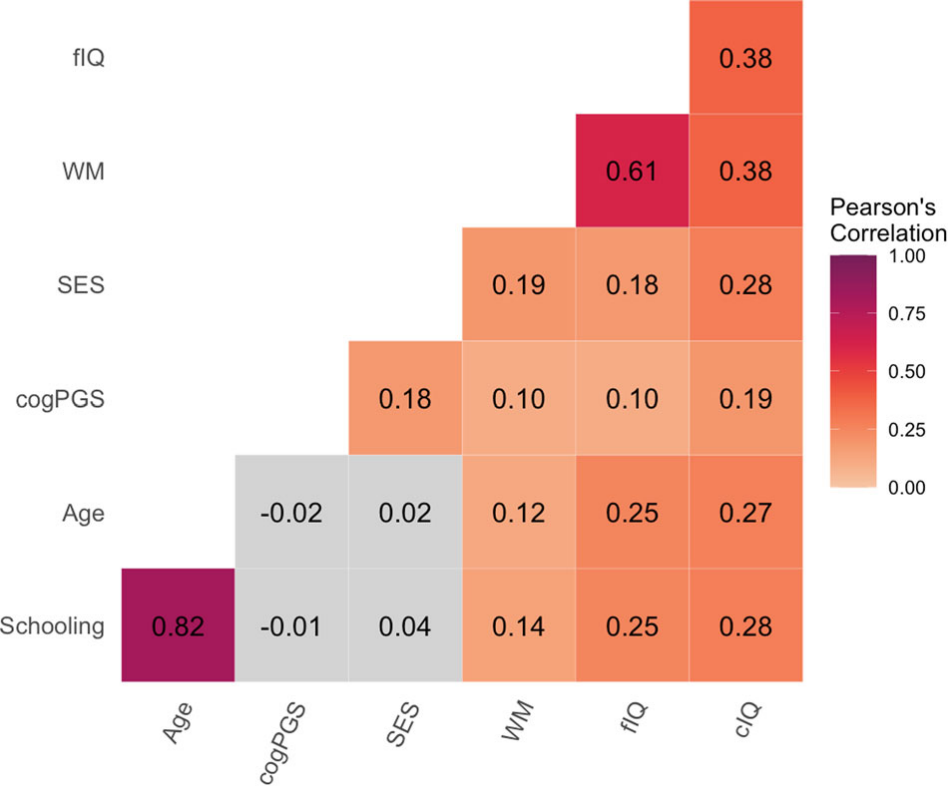
Corrected correlation plot. A correlation plot showing the relationships of our variables of interest, colored values indicate *p* *<* 0.001. These values are corrected for genetic PC’s (see Supplementary Fig. 4 for more variables).

We included data from 6567 children (mean age = 9.88, range 8.92–11.00; Supplementary Table 1) recruited by the ABCD consortium to be representative of the United States in sex, race, ethnicity, SES, and urbanicity^47^. SES was defined as the first component of a probabilistic PCA, capturing 65% of the variance in total household income, highest parental education, and neighborhood quality. Due to modeling constraints, we had to exclude 1,086 children that were missing DNA data. This group had significantly lower SES (Cohen’s *d* = −0.23, *p* < 0.001). SES and cogPGS were positively correlated with each other and each cognitive domain (Fig. 1 and Supplementary Fig. 4).

### Schooling, SES, and cogPGS effect on cognition

To examine the effect of schooling on *c*IQ, *f*IQ, and WM development, we used a regression discontinuity design over grades 3 to 5, while controlling for age, sex, cogPGS, SES, and 20 ancestry-based principal components (Eq. 2, Supplementary Tables 3–5).

**Fig. 2:**
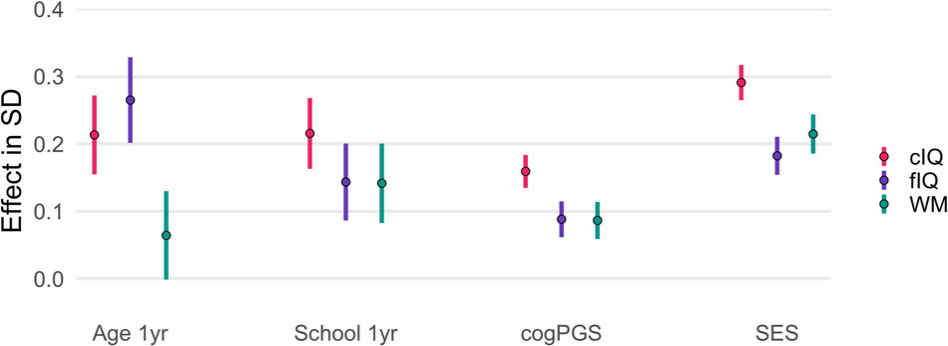
Main effects for cognition. Independent effects of 1 year of age, 1 year of schooling, cogPGS, and SES from mixed-effects models predicting *c*IQ, *f*IQ, and WM (Eq. 2). All results are significant for predicting their respective cognitive domain (Supplementary Tables 3–5). Bars reflect 95% frequentist confidence intervals.

We found that schooling had a significant effect (all *p*’s < 0.001) for each cognitive domain we examined (*c*IQ: *β* = 0.13, *f*IQ: *β* = 0.10, WM: *β* = 0.09). One year of schooling (i.e., 10 months) contributed 0.22 SD, 0.14 SD, and 0.14 SD to the development of cIQ, fIQ, and WM, respectively (Fig. 2). Notably, the ratio of 1 year of schooling to 1 year of chronological age differed depending on the domain. As expected, schooling affected *c*IQ (ratio = 1.1) more than *f*IQ (ratio = 0.54). Interestingly, WM, a subtask of *f*IQ, had the highest ratio of 2.2, indicating for this age range, 1 year of schooling had double the effect of 1 year of chronological age. There were no significant differences between the schooling coefficients for *c*IQ, *f*IQ, or WM (|Z| < 1.96).

Sex had a significant effect for each cognitive domain, with females performing better in *f*IQ (*β* = 0.09, *p* < 0.001) yet worse in *c*IQ (*β* = −0.05, *p* = 0.023) and WM (*β* = −0.08, *p* < 0.001). CogPGS and SES both showed independent significant effects (all *p’*s < 0.001) for *c*IQ, *f*IQ, and WM. The effect sizes of cogPGS (*β* = 0.16) and SES (*β* = 0.29) were the largest for *c*IQ. CogPGS showed similarly sized effects for *f*IQ (*β* = 0.09) and WM (*β* = 0.09), yet SES had a slightly higher effect for WM (*β* = 0.22) than *f*IQ (*β* = 0.18).

### SES subcomponents

The analysis above showed that our combined measure of SES had effects on *c*IQ, *f*IQ, and WM. Since research has shown that different subcomponents of SES could relate differently to intelligence^48–50^, we also performed an analysis of each subcomponent. All components were significant (*p*_FDR_ < 0.001) for each cognitive domain. *c*IQ showed the largest effects for parental education (*β* = 0.26), family income (*β* = 0.22) and neighborhood quality (*β* = 0.11). In a similar fashion to the SES composite, WM had slightly larger effect sizes than *f*IQ for parental education (WM: *β* = 0.19; *f*IQ: *β* = 0.17), family income (WM: *β* = 0.17; *f*IQ: *β* = 0.14), and neighborhood quality (WM: *β* = 0.08; *f*IQ: *β* = 0.06).

### Sibling analysis

Gene-environment correlations can inflate the estimation of cogPGS on phenotypes^33,51,52^. Therefore, we conducted a post hoc sibling analysis (families = 392, *n* = 792) to estimate within (*β*_*w*_) and between (*β*_*B*_) family effects of cogPGS on *c*IQ, *f*IQ, and WM. Within-family effects (*β*_*w*_) are less confounded by the shared environment since the transmission of alleles is random, giving each sibling an equal probability of inheriting any given allele. Yet, there is no variance within families for SES and very little for schooling therefore, the sibling analysis is limited to genetics (cogPGS). We found *c*IQ’s within-family effect (*β*_*w*_ = 0.08, *p*_FDR_ = 0.009) to be roughly half of the between-family effect (*β*_*B*_ = 0.15, *p*_FDR_ = 0.009). For *f*IQ we found a significant within-family effect (*β*_*w*_ = 0.08, *p*_FDR_ = 0.013), yet the between-family effect was not significant (*β*_*B*_ = 0.10, *p*_FDR_ = 0.074). For WM, neither the within-family (*β*_*w*_ = 0.04, *p*_FDR_ = 0.235) nor the between-family (*β*_*B*_ = 0.10, *p*_FDR_ = 0.066) effects were significant.

### Gene-by-environment interplay

**Fig. 3:**
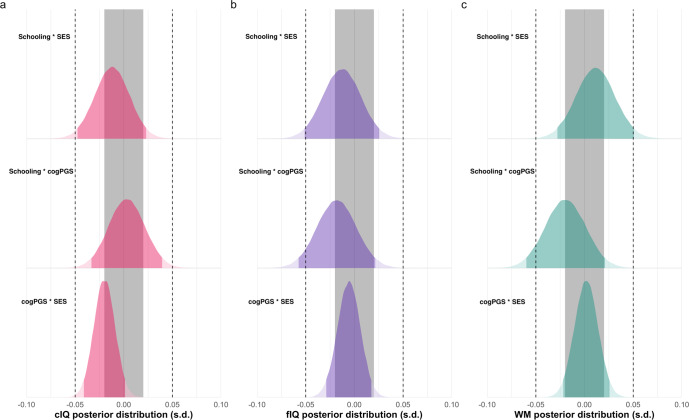
Interaction effects for cognition. The estimated interaction effects of schooling-SES, schooling-cogPGS and cogPGS-SES on **a** cIQ, **b** fIQ, and **c** WM from a hierarchical Bayesian mixed-effects model. All values are in standard units. Dark blue shading indicates the 95% highest-density interval’s (HDI) of the estimates posterior distribution. The gray band represents a region of practical equivalence (ROPE) of 0.02 SD’s, while the dotted lines indicate a ROPE boundary of 0.05 SD’s. Most of the interaction terms are fully within a 0.05 SD ROPE boundary providing evidence for the null hypothesis at this boundary, yet none fully lie within a ROPE boundary of 0.02 SD’s (Supplementary Table 8).

There were no significant interactions for any of the cognitive domains (Eqs. 3 and 4; Supplementary Tables 3–5). This included our two-way interaction terms of interest (i.e., schooling-cogPGS, schooling-SES, and cogPGS-SES) and the three-way interaction term (i.e., schooling-cogPGS-SES). This null result held when we added principal component interactions with schooling, cogPGS, and SES for each model^53^. We, therefore, carried out post hoc Bayesian null hypothesis testing using region of practical equivalence (ROPE) boundaries with 95% highest-density intervals (HDI) for the two-way interaction terms of interest (Fig. 3, Eq. 3). We used weakly informative priors, centered around zero, for the two-way interaction terms (see Methods). Crucially, we used two ROPE boundaries in standard units of 0.05 and 0.02. This was done for two reasons, (1) a lack of consensus in the field on a minimal effect size of interest and (2) schooling representing a cumulative process^54–56^.

Almost all interaction terms were entirely within a ROPE boundary of 0.05 (Fig. 3). This means that with a 95% probability, the interaction effect was less than 0.05 SD, adding evidence for the null hypothesis (Supplementary Table 8). The two exceptions were the schooling-cogPGS interaction (Fig. 3b, c), where 95% were slightly outside the 0.05 boundary for *f*IQ (3%) and WM (5%). However, most of the terms have a substantial part of their distribution overlapping with the ROPE boundaries of 0.02. This means we cannot confirm nor disprove the null hypothesis for a maximal effect size of interest of 0.02 SDs.

### Post hoc *g* analysis

A single-factor confirmatory factor analysis fit the cognitive tasks well (RMSEA *=* 0.03 & CFI *=* 0.987; Supplementary Fig. 2). We then extracted factor scores (*g*) and used them as the dependent variable. The standardized effect of schooling on *g* was 0.136 SD (*p*_FDR_ *<* 0.001)—a similar magnitude as fIQ and WM’s effects. Mirroring the other cognitive domains, no two- or three-way interactions were significant following FDR correction (Supplementary Table 6).

### Post hoc European ancestry analysis

CogPGS has less accuracy in non-European populations therefore, we conducted a post hoc reanalysis in the full model (Eq. 4) for each cognitive variable, including only those children whose genetic ancestry 4-means clustered on the first two principal components^57,58^. This subsample was less representative of the United States with substantially higher SES (Cohen’s *d* *=* 0.80., *p* *<* 0.001). The results from that analysis stayed generally the same as the analysis of the full population (Supplementary Table 7), both in terms of effect sizes on the main results and the lack of significance on the interaction terms. In reference to the full sample, there was a slight increase in the size of the effect from CogPGS (β range *=* 0.005–0.020), yet this was followed by larger decreases from SES (β range = −0.046 to −0.080).

## Discussion

Schooling showed substantial and independent effects for each intelligence domain tested: *c*IQ, *f*IQ, and WM. In line with previous research, we found the raw effect of schooling on *c*IQ to be larger than for *f*IQ, though this difference was not significant^13^. This was also true for the relative influence of schooling compared to the effect of chronological age, as that ratio for *c*IQ was almost double that of *f*IQ (1.1 vs 0.54), showing almost near equal influences per year of schooling and age. Surprisingly, WM had the highest ratio (2.2), with the effect of schooling being more than double that of age. While this is in agreement with a previous study in younger children^21^, it should be interpreted with caution as the WM measure comprised of only one task^59^.

As expected, SES and cogPGS were highly correlated with each other, highlighting the need to isolate the independent effects of each. Both had large, independent effects on *c*IQ, *f*IQ, and WM. In a follow-up analysis, we estimated the contribution of each SES component separately. Notably, all SES components were significant for each intelligence domain, with the effects from parental education and income being similar in size while neighborhood quality was roughly half.

A child’s SES is not independent of their cogPGS^31^, which makes it difficult to support causal inferences of these factors as well as interpret the interaction between them (i.e., the endogeneity problem)^60^. This gene-environment dependence can cause spurious gene-environment interactions^41,61^. Our sibling analysis sheds light on this issue. We found the within-family effect of cogPGS to be roughly half of the between-family effect for *c*IQ, in line with the literature^51,52^. This indicates the presence of passive genotype-environment correlations—whereby parents create family environments consistent with their genotypes, which in turn facilitate the development of their children’s intelligence. Since we only had data from 392 families, a lack of statistical power is most likely the reason for our null findings for *f*IQ and WM. While our cogPGS estimate in the full sample should be interpreted with caution, previous research has shown SES to be the major source of these between-family effects^51,62^.

Predominant theories of GE-interplay imply a positive sign—genetically endowed cognition influences one’s proximal environment, and that environment, in turn, influences one’s cognition in continuous, reciprocal interactions, such as the multiplier theory^34^, the transactional model^35^ and the bioecological model^36^. In line with this, a meta-analysis on twin research found the heritability of intelligence to increase with higher SES in the United States^63^. But this effect is far from consistent. The same meta-analysis did not replicate this finding on data collected outside the United States. Furthermore, a large twin study found negative and null results for heritability by SES interaction regarding mathematics and reading in Florida^64^. Two studies using a similar polygenic marker to ours did not find evidence of GE-interplay for EA^65,66^, yet a study with 130,000 adults in the UK found a very small negative interaction of SES with neighborhood quality for *f*IQ and EA^40^. Crucially, the standardized effect size of this interaction was (*β* < 0.02 SD)—translating to less than a third IQ point throughout one’s entire life—in turn having no practical effect for the individual.

We did not find any significant interaction between schooling and SES or cogPGS. One strength of our design is that schooling is, in principle, independent from cogPGS and SES. We are not aware of any other research looking at GE-interplay with schooling. A recent study did find low PGS children in high-SES schools to continue with mathematics much longer than genetically similar children in low-SES schools^39^. However, we see their result as more relevant to inform a cogPGS-SES effect rather than the gene-by-schooling interaction.

We expected our interaction terms to either compensate or accelerate preexisting differences. Schooling, for example, could increase (i.e., Mathew’s effect) economic/genetic inequality or lessen these differences between children (i.e., catch-up effect). The Coleman Report, a seminal study with more than half a million students and over 3000 schools in 1966, controversially concluded schools did not contribute to widening achievement gaps between children^29^. Conversely, there is some evidence that schooling might lessen socioeconomic disparities between children (i.e., a catch-up effect) for cognitive skills^67^.

Our study indicates schooling to not be a major driving force for either increasing or decreasing differences due to SES or cogPGS. Yet, we emphasize caution in interpreting these null effects as our range of schooling (3rd–5th grade) was limited, and Bayesian analysis showed that an effect of less than 0.02 SD could not be ruled out (Fig. 3). Since any interaction effect with schooling could accumulate—that is, continue to increase each year—a very small (<0.05 SD) effect size could be of practical relevance^54,55^. For example, an interaction with schooling as small as 0.02 SD could accumulate over five years to 0.1 SD or roughly 30% of the largest SES main effect (i.e., *c*IQ = 0.29). This is, of course, a simplistic scenario assuming no counteracting mechanisms, yet it illustrates how very small effect sizes can become consequential^68,69^. In contrast, cogPGS–SES’ interaction is a lifelong effect and does not have the potential to accumulate in the same way. However, our sample had a slightly lower SES (Cohen’s *d* = −0.23) than the average for the United States, therefore, we cannot rule out an interaction at the lower tail of the SES distribution.

One limitation of this study is that the 1.1 million individuals used to estimate the cogPGS are heavily biased towards those of European descent and from higher SES areas^30,70^. This means our results regarding genetics should only be generalized to white populations. Furthermore, GWAS methods cannot detect certain types and sizes of GE interactions since they are intended to detect additive effects^71^. Another consideration is how to interpret findings with multi-trait GWAS’s—in our case cognitive ability, mathematics, and educational attainment—since one of the supplementary phenotypes could be driving the results^72^. The extent of this issue depends on (1) the relative sample size differences between the GWAS included and (2) the genetic correlation of these traits. In our case, there are sample size differences between educational attainment and cognitive ability, yet the very high (r ~ 0.75) genetic correlation between these traits most likely mitigates this issue^73^. Lastly, a strength of our study is that we controlled for ancestry-based genetic PCs in the full model, rather than just correcting cogPGS. While this means SES’s relationship with cognition is controlled for population stratification, it also brought the limitation that we had to exclude subjects without DNA, resulting in the average level of SES increasing.

We found that schooling causes relatively large increases in children’s intelligence. The two years of schooling (3rd to 5th grade) caused a larger difference in intelligence than either SES or cogPGS. However, schooling did not change the rank order of individuals’ intelligence. This was shown by the lack of significant two-way interactions between Schooling, SES, and cogPGS, although our power to detect potentially meaningful small effects for schooling was limited. Intriguingly, we did not find any interaction between SES and cogPGS, this means that children’s genetic differences do not matter more, or less, for intelligence dependent upon their SES background.

## Methods

We used data from the Adolescent Brain Cognitive Development (ABCD) study, which was reviewed and approved by the central institutional review board at the University of California, San Diego. Written parental informed consent, along with child assent, was obtained for all participants. Part of the sample included siblings, along with non-representative amounts of monozygotic and dizygotic twins^47,74^. To correct this, we randomly selected one child per family. We also excluded children that reported ever repeating a grade, as this would bias our statistical analysis. This resulted in a total of 6567 children between grades three and five enrolled in regular private or public schooling.

### Behavioral and demographic measures

Since recruitment was continuous throughout the year, this allowed us to measure schooling in months. We excluded summer vacation (15 June until 15 August) from our schooling variable by coding children recruited during summer as having the last month of schooling from the previous grade. This resulted in a variable with 10 months of schooling per year. Chronological age was also measured in months to preserve anonymity.

Our endogenous variables were working memory (WM), fluid (*f*IQ), and crystallized intelligence (*c*IQ) from the NIH toolbox cognition battery^75^. These tasks have been shown to have good reliability and validity^59,76,77^. *c*IQ consisted of a picture vocabulary task and an oral reading recognition task. *f*IQ consisted of five tasks; pattern comparison processing speed test, a list-sorting WM test, a picture sequence memory test, a flanker task, and a dimensional change card sort task. One of the fluid tasks, list-sorting WM, was used as our WM measure. We used the sum scores provided by the NIH toolbox, rather than latent factors, to facilitate comparison with other studies. All outliers were brought to the fence (Tukey/Boxplot Method). Variables were then standardized to a mean of zero and a standard deviation of one.

SES was defined as the first principal component from a probabilistic PCA of total household income, highest parental education, and neighborhood quality. We used a probabilistic PCA because of the decent amounts of non-overlapping missing data in parental income (*n* = 474, ~7%) and neighborhood quality (*n* = 263, ~4%). Children missing more than one of the three measures were excluded (*n* = 45). Parental education was recorded to reflect middle school or less (1), some high school (2), high school graduate (3), some college/associates degree (4), bachelor’s degree (5), a master’s degree (6), or professional degree (7). Our measure of neighborhood quality was the area deprivation index calculated from the American Community Survey using the address of primary residency^78^. The SES composite and each subcomponent were standardized with a mean of zero and a standard deviation of one.

### Genetic measures

Genotyping was done by the ABCD study and the data provided to us. Saliva samples were collected at the baseline visit the genotyping was performed using the Smokescreen array^79^, consisting of 646,247 genetic variants.

Quality control (QC), imputation, and genetic PCA were performed by the National Bioinformatics Infrastructure Sweden (NBIS), as a service contracted by us. Before imputation, SNPs were excluded if they had high levels of missing data (SNP call rate <98%), departed from Hardy–Weinberg equilibrium as calculated in the lfa R package (sHWE) (*P* < 1 × 10^−6^), or had minor allele frequencies (MAF) <1%. Moreover, individuals with an absolute autosomal heterozygosity >0.2 or more than 2% missing genotypes were excluded. These filtering steps resulted in a cleaned dataset of 10,069 individuals and 430,622 variants. Subsequently, haplotypes were pre-phased with SHAPEIT2. Genetic markers were imputed using the IMPUTE4 software. As the reference population, we used the 1000 Genomes haplotypes—Phase 3 integrated variant set release in NCBI build 37 (hg19) coordinates. This is a mixed, multi-ethnic population dataset consisting of 2504 samples and 5008 haplotypes from populations of Europeans, Africans, East Asians, Southern Asians, and Americans (https://mathgen.stats.ox.ac.uk/impute/1000GP_Phase3.html). One of the main advantages of this imputation approach is that it provides better concordance in diverse human populations (for more information, see refs. ^80,81^). After imputation, genotypes with an INFO score <0.3 or a MAF <0.001% were excluded. The final number of SNPs after imputation was 40,637,119 in a total of 10,069 individuals.

To check for outliers and to control for population structure, principal components of SNPs were obtained using the principal component analysis (PCA) module as implemented in RICOPILI. First, SNPs were pruned to ensure that there is little linkage disequilibrium between SNPs (R2 < 0.2, 200 SNPs window: plink – indep-pairwise 200 100 0.2). The LD pruning was repeated until 100 K SNPs were reached. Then, the resulting SNPs go into the PCA. We used the first 20 principal components (PCs) from that genetic PCA as covariates in our main analyses.

With the SNPs QCed and imputed, our group then created polygenic scores for cognitive performance (here called “cogPGS”) for each participant using PRSice-2^82^. This was calculated by the sum of effect sizes of thousands of SNPs (weighted by how many of the effect alleles were present in each individual) that were discovered by a large genome-wide association study on educational attainment, mathematical ability, and general cognitive ability^30^. That study has available all effects sizes and *p* values of their SNPs on the website of the Social Science Genetics Association Consortium (https://www.thessgac.org/data). We used the data available by the consortium from a multi-trait analysis of GWAS^83^, which, in our case, represents a joint polygenic score focused on a GWAS of cognitive performance and complemented by information from a GWAS on educational attainment, a GWAS on the highest-level math class completed, and a GWAS on self-reported math ability. This joint analysis is ideal because pairwise genetic correlations of these traits were high^30^. Furthermore, these GWAS had hundreds of thousands of individuals, and such a large sample size allows new studies to detect effects in samples of a few hundred individuals with 80% statistical power^30^.

For the creation of cogPGS in our samples, we performed clumping and pruning to remove nearby SNPs that are correlated with one another. We used the SNPs below the original threshold of *P* < 5e-5 from the GWAS as we wanted to use the full GWAS sample (limited SNP release due to anonymity concerns). After adopting a clumping sliding window of 250 kb, with the linkage disequilibrium clumping set to *r*^2^ > 0.25, this resulted in 5255 SNPs for our dataset. Finally, we normalized the polygenic scores (mean = 0, sd = 1).

### Statistical analysis

We used random intercept mixed-effects models to predict the effect of schooling from our three cognitive variables of interest (i.e., *f*IQ, *c*IQ, and WM); Y denotes these outcome variables. Every model included a random intercept per collection site (*j*) to account for the clustering of individuals (*i*). Models were fit using maximum likelihood estimation with the R package lme4 (v. 1.1–27.1)^84,85^. All results are reported in standard units (mean = 0, SD = 1), unless specified otherwise. *P* values on all models were derived using Satterthwaite’s degrees of freedom method with the lmerTest package (v. 3.1-3)^86^, with an alpha level of 0.05.

We broke the equation into four steps to more clearly convey the identification assumptions behind our estimands^87^. A fuzzy regression discontinuity design (Eq. 1) was used to isolate the effect of one year of schooling from chronological age. This causal inference method is one of the best to isolate the effect of schooling, yet it relies on two critical assumptions: (1) age-based allocation rule and (2) linearity of the within-grade regression. Previous research has shown that, in practice, violation of these assumptions does not substantially bias the schooling or age coefficient^18,88^. Data collection was continuous throughout the school year therefore, we correct for months of schooling rather than grade, yet our instrument (age-based grade allocation) is on the level of grade. We included post hoc sensitivity checks using grades 3–5 and comparing the two most frequent, grades 4 and 5, for Eq. 1 (Supplementary Table 9a, b). Lastly, we also compared the schooling coefficients using a *z*-test on the difference between the coefficients. This method sums the standard error from the coefficients as error variance, assuming no correlation between the coefficients, thereby being conservative^89^. We used an absolute z-value of 1.96, corresponding to an alpha level of 0.05.

1 $$y_{ij} \sim {\upalpha}_0 + {\upbeta}_1( {{\mathrm{Age}}_{ij}} ) + {\upbeta}_2( {{\mathrm{Schooling}}_{ij}} ) + {\upgamma}_{0j} + \epsilon _{ij}$$

We then add a fixed effect for cogPGS and SES, while controlling sex and 20 genetic principal components (Eq. 2). β_*_ represents 20 fixed effects for each principal component. This model allowed us to estimate the causal effect of one year of schooling, along with estimating the independent effects of chronological age, sex, cogPGS, and SES. Crucially, schooling is the only exogenous variable. It is important to highlight that by adding 20 genetic principal components to the model, we are controlling all variables for population stratification, most notably SES’s relationship with intelligence.

2 $$\begin{array}{l}y_{ij} \sim \alpha _0 + \beta _1( {{\mathrm{Age}}_{ij}} ) + \beta _2( {{\mathrm{Shooling}}_{ij}}) + \beta _3( {{\mathrm{SES}}_{ij}} ) + \beta _4( {{\mathrm{cogPGS}}_{ij}} )\\ \qquad\quad\;\;\,+\, \beta _5( {{\mathrm{SexMale}}_{ij}} ) + \beta _ \ast ( {20{\mathrm{PC}}_{ij}} ) + \gamma _{0j} + {\it{\epsilon }}_{ij}\end{array}$$

In Eq. 3, we were interested in determining if schooling interacted with cogPGS (i.e., *β*_6_) or SES (i.e., *β*_7_). To properly isolate schooling’s interaction effects and avoid spurious results, we included interactions of no interest with age (i.e., *β*_8_ and *β*_9_). We were also interested to see if SES interacted with cogPGS (i.e., *β*_10_), yet we emphasize caution in interpreting this term as any interaction found could be spurious due to endogeneity between SES and cogPGS^41,90^. We refer to these interactions as GE-interplay, highlighting that they could be caused by gene-by-environment interaction (GxE) and/or gene-by-environment correlation (rGE). rGE refers to the fact that genotypes and environments are not randomly distributed, while GxE is an interaction in the classical sense whereby the environment (or genotype) interacts with the other^91^. Due to schooling’s exogenous nature, a significant interaction would be most likely due to GxE, yet this is not the case for an interaction between SES and cogPGS which could be the result of either GxE or rGE.

3 $$\begin{array}{ll} y_{ij} \sim \alpha _0 + \beta _1( {{\mathrm{Age}}_{ij}} )+ \beta _2( {{\mathrm{Schooling}}_{ij}} )\beta _3( {{\mathrm{SES}}_{ij}} ) + \beta _4( {{\mathrm{cogPGS}}_{ij}} )\\ \qquad\quad\;\;\,+ \,\beta _5( {{\mathrm{SexMale}}_{ij}} ) + \beta _6( {{\mathrm{Schooling}} \times {\mathrm{cogPGS}}_{ij}} )\beta _7( {{\mathrm{Schooling}} \times {\mathrm{SES}}_{ij}} ) \\ \qquad\quad\;\;\,+\,\beta _8( {{\mathrm{Age}} \times {\mathrm{cogPGS}}_{ij}}) + \beta _9( {{\mathrm{Age}} \times {\mathrm{SES}}_{ij}} )\\ \qquad\quad\;\;\,+\, \beta _{10}( {{\mathrm{cogPGS}} \times {\mathrm{SES}}_{ij}} ) + \beta _ \ast ( {20{\mathrm{PC}}_{ij}} ) + \gamma _{0j} + \epsilon _{ij} \end{array}$$

Lastly, we were interested in the presence of a three-way interaction between schooling, SES, and cogPGS (Eq. 4, *β*_11_). To accomplish this, we added a three-way interaction term, which was then compared, using a likelihood-ratio test to Eq. 3.

4 $$\begin{array}{l} y_{ij} \sim \alpha _0 + \beta _1( {{\mathrm{Age}}_{ij}}) + \beta _2( {{\mathrm{Schooling}}_{ij}}) + \beta _3( {{\mathrm{SES}}_{ij}} ) + \beta _4( {cogPGS_{ij}} ) \\\qquad\quad\;\;\,+\, \beta _5( {{\mathrm{SexMale}}_{ij}} ) + \beta _6( {{\mathrm{Schooling}} \times {\mathrm{cogPGS}}_{ij}} ) \\\qquad\quad\;\;\,+\, \beta _7( {{\mathrm{Schooling}} \times {\mathrm{SES}}_{ij}} ) + \beta _8( {{\mathrm{Age}} \times {\mathrm{cogPGS}}_{ij}} ) \\\qquad\quad\;\;\,+\, \beta _9( {{\mathrm{Age}} \times {\mathrm{SES}}_{ij}} ) + \beta _{10}( {{\mathrm{cogPGS}} \times {\mathrm{SES}}_{ij}} ) \\\qquad\quad\;\;\,+\, \beta _{11}( {{\mathrm{Schooling}} \times {\mathrm{cogPGS}} \times {\mathrm{SES}}_{ij}}) + \beta _ \ast ( {20{\mathrm{PC}}_{ij}} ) + \gamma _{0j} + {\it{\epsilon }}_{ij} \end{array}$$

### SES subcomponents

In cases of significant SES composite findings, we also reported the results for each SES subcomponent separately. This was done for two reasons: (1) research showing specific SES measures to relate to intelligence more than others, and (2) call in the literature to treat SES measures independently^48–50^. We considered the entire subcomponent analysis a family, therefore, *p* values were corrected using a false discovery rate (FDR) for the number of terms tested.

### Post hoc sibling analysis

To estimate the within and between-family effect cogPGS on *c*IQ, *f*IQ, and WM we conducted a sibling analysis using the procedure described by Selzam and colleagues 2019^51^. This allowed us to estimate the within-family direct genetic effects (*β*_*w*_) and the between-family genetic effects (*β*_*B*_). For families to be included in the analysis they needed to have at least two non-identical siblings. We considered the entire sibling analysis a family; therefore, *p* values were corrected using false discovery rate (FDR) for the number of terms of interest tested. SES has been shown to largely explain between-family effects therefore cogPGS in our main analysis is, to some extent, controlled for these between-family effects^51,62^.

### Post hoc *g* analysis

We fit nine cognitive tasks into a single-factor solution using confirmatory factor analysis with the Lavaan package in R (Supplementary Fig. 2)^92^. This included all the tasks from the NIH toolbox cognition battery along with the overall score on a WM nback task and the total raw score from matrix reasoning on the Wechsler Intelligence Scale for Children. A single factor solution fit the data well (RMSEA = 0.03 and CFI = 0.987) when correlated covariances were added between the two crystallized tasks, the two WM tasks, and a cluster of three tasks sharing similar attributes (Flanker, Pattern comparison & Card sorting). We then extracted factor scores and fit them to Eqs. 1–4 while using FDR correction for multiple comparisons.

### Post hoc European ancestry analysis

CogPGS has limited prediction accuracy in non-European samples^57^. We therefore, subsetted the data using 4-means clustering on the first two principal components (*n* = 3751), resulting in the exclusion of 43% of the sample^58^. The proportion is equal to the full ABCD sample, which is representative of the United States. Yet this subsample had significantly higher SES (Cohen’s *d* = 0.80., *p* < 0.001). All variables were (re)standardized to this population with a mean of 0 and an SD of 1. We then fit Eq. 4 on this subset for *c*IQ, *f*IQ, WM, and *g* and used FDR correction on the *p* values (Supplementary Table 7).

### Bayesian hierarchical modeling

To have a better understanding of our results, we conducted Bayesian mixed-effects models of Eq. 3 with the brms package (v. 2.15.0)^93^ using Markov Chain Monte Carlo sampling with 10,000 iterations (1000 warmup) in 15 chains. We set weakly informative, normally distributed priors for our terms of interest, keeping the default non-informative priors unless otherwise specified. For *c*IQ age (*β*_1_) and schooling (*β*_2_) had normally distributed priors with a mean of .20 and a standard deviation (SD) of 0.15. CogPGS (*β*_4_) had a prior with a mean of 0.15 and an SD of 0.1, while SES (*β*_3_) had a larger mean of 0.25 and an SD of 0.2. All two-way interaction terms (*β*_6-10_) had two-tailed priors with a mean of zero and an SD of 0.1. Gender and the 20 PCs had default flat priors. For *f*IQ and WM we set identical priors; these were a mean of 0.15 and an SD of 0.15 for age (*β*_1_) and schooling (*β*_2_). CogPGS (*β*_4_) had a prior with a mean of 0.1 and an SD of 0.1, while SES (*β*_3_) was double that with a mean of 0.2 and an SD of 0.2. All two-way interaction terms (*β*_6-10_) had identical priors to *c*IQ, along with flat priors for gender and each principal component. We also fit Eq. 4 to test the null of the three-way interaction term where priors were identical to Eq. 3 while the three-way term (*β*_11_) had a mean of 0 and an SD of 0.1. Notably, our results for the interactions in Eqs. 3 and 4 did not meaningfully change in comparison to flat priors.

We then computed the 95% highest-density intervals (HDI) of the posterior distributions for our three interaction terms of interest (*β*_6_, *β*_7_, *β*_10_, *β*_11_) for *c*IQ, *f*IQ, and WM. Crucially, we used two ROPE boundaries in standard units of 0.05 and 0.02. This was done for two reasons, (1) a lack of consensus in the field on a minimal effect size of interest, and (2) schooling representing a cumulative process. Since the schooling interaction terms (*β*_6_, *β*_7_, *β*_11_) are cumulative, they could warrant a smaller minimal effect size of interest than the cogPGS-SES’ interaction^54–56^. We then calculated the percentage of the estimated 95% HDI lying within our ROPE boundaries, giving us the probability of the null hypothesis. If 95% of the estimates' posterior distribution lies within ROPE boundaries, the null hypothesis can be confirmed as any probable effect is considered too small to be meaningful.

### Reporting summary

Further information on research design is available in the Nature Research Reporting Summary linked to this article.

## Supplementary information

Supplementary Material

Reporting Summary

## Data Availability

The data to replicate this analysis is on the NIHM data archive (https://nda.nih.gov) under release 3.0 “ABCDstudyNDA”.

## Code availability

The code to replicate all analysis and figures will be made available upon publication at https://github.com/njudd/ABCDschooling.

## References

[1] Calvin CM (2017). Childhood intelligence in relation to major causes of death in 68 year follow-up: prospective population study. BMJ.

[2] Batty GD (2009). IQ in early adulthood and mortality by middle age: cohort study of 1 million Swedish men. Epidemiology.

[3] Deary, I. J., Cox, S. R. & Hill, W. D. Genetic variation, brain, and intelligence differences. *Mol. Psychiatry*10.1038/s41380-021-01027-y (2021). 10.1038/s41380-021-01027-y PMC8960418 33531661

[4] Deary IJ, Strand S, Smith P, Fernandes C (2007). Intelligence and educational achievement. Intelligence.

[5] Frey MC, Detterman DK (2004). Scholastic assessment or g? The relationship between the Scholastic Assessment Test and general cognitive ability. Psychol. Sci..

[6] Flynn JR (1987). Massive IQ gains in 14 nations: what IQ tests really measure. Psychol. Bull..

[7] Scarr S, McCartney K (1983). How people make their own environments: a theory of genotype–> environment effects. Child Dev..

[8] van Ijzendoorn MH, Juffer F, Poelhuis CWK (2005). Adoption and cognitive development: a meta-analytic comparison of adopted and nonadopted children’s IQ and school performance. Psychol. Bull..

[9] Polderman TJC (2015). Meta-analysis of the heritability of human traits based on fifty years of twin studies. Nat. Genet..

[10] Davies G (2011). Genome-wide association studies establish that human intelligence is highly heritable and polygenic. Mol. Psychiatry.

[11] Bouchard TJ (2009). Genetic influence on human intelligence (Spearman’s g): how much?. Ann. Hum. Biol..

[12] Haworth CMA (2010). The heritability of general cognitive ability increases linearly from childhood to young adulthood. Mol. Psychiatry.

[13] Ritchie SJ, Tucker-Drob EM (2018). How much does education improve intelligence? A meta-analysis. Psychol. Sci..

[14] Ceci SJ (1991). How much does schooling influence general intelligence and its cognitive components? A reassessment of the evidence. Dev. Psychol..

[15] Lazar, I. et al. Lasting effects of early education: a report from the consortium for longitudinal studies. *Monogr. Soc. Res. Child Dev*. **47**, 1–151 (1982).

[16] Clouston SAP (2012). Benefits of educational attainment on adult fluid cognition: international evidence from three birth cohorts. Int. J. Epidemiol..

[17] Brinch CN, Galloway TA (2012). Schooling in adolescence raises IQ scores. Proc. Natl Acad. Sci. USA.

[18] Luyten H (2006). An empirical assessment of the absolute effect of schooling: regression‐discontinuity applied to TIMSS‐95. Oxf. Rev. Educ..

[19] Cahan S, Cohen N (1989). Age versus schooling effects on intelligence development. Child Dev..

[20] Baltes PB, Reinert G (1969). Cohort effects in cognitive development of children as revealed by cross-sectional sequences. Dev. Psychol..

[21] Roberts G (2015). Schooling duration rather than chronological age predicts working memory between 6 and 7 years: Memory Maestros Study. J. Dev. Behav. Pediatr..

[22] Lubinski D (2009). Cognitive epidemiology: with emphasis on untangling cognitive ability and socioeconomic status. Intelligence.

[23] Tucker-Drob EM (2013). How many pathways underlie socioeconomic differences in the development of cognition and achievement?. Learn. Individ. Differ..

[24] von Stumm S, Plomin R (2015). Socioeconomic status and the growth of intelligence from infancy through adolescence. Intelligence.

[25] von Stumm S (2020). Predicting educational achievement from genomic measures and socioeconomic status. Dev. Sci..

[26] Hart SA, Little C, van Bergen E (2021). Nurture might be nature: cautionary tales and proposed solutions. NPJ Sci. Learn.

[27] Smith-Woolley E (2018). Differences in exam performance between pupils attending selective and non-selective schools mirror the genetic differences between them. NPJ Sci. Learn.

[28] Trzaskowski M (2014). Genetic influence on family socioeconomic status and children’s intelligence. Intelligence.

[29] Coleman, J. S. et al. The coleman report. *Equality of Educational Opportunity* (1966).

[30] Lee JJ (2018). Gene discovery and polygenic prediction from a genome-wide association study of educational attainment in 1.1 million individuals. Nat. Genet..

[31] Abdellaoui A (2019). Genetic correlates of social stratification in Great Britain. Nat. Hum. Behav..

[32] Judd N (2020). Cognitive and brain development is independently influenced by socioeconomic status and polygenic scores for educational attainment. Proc. Natl Acad. Sci. USA.

[33] Sauce B, Matzel LD (2018). The paradox of intelligence: heritability and malleability coexist in hidden gene-environment interplay. Psychol. Bull..

[34] Dickens WT, Flynn JR (2001). Heritability estimates versus large environmental effects: the IQ paradox resolved. Psychol. Rev..

[35] Tucker-Drob EM, Briley DA, Harden KP (2013). Genetic and environmental influences on cognition across development and context. Curr. Dir. Psychol. Sci..

[36] Bronfenbrenner U, Ceci SJ (1994). Nature-nuture reconceptualized in developmental perspective: a bioecological model. Psychol. Rev..

[37] Trejo S (2018). Schools as moderators of genetic associations with life course attainments: evidence from the WLS and Add Heath. Sociol. Sci..

[38] Schmitz LL, Conley D (2017). The effect of Vietnam-Era conscription and genetic potential for educational attainment on schooling outcomes. Econ. Educ. Rev..

[39] Harden, K. P., Domingue, B. W. & Belsky, D. W. Genetic associations with mathematics tracking and persistence in secondary school. *NPJ Sci. Learn.* **5**, 1 (2020). 10.1038/s41539-020-0060-2 PMC7002519 32047651

[40] Rask-Andersen, M., Karlsson, T., Ek, W. E. & Johansson, Å. Modification of heritability for educational attainment and fluid intelligence by socioeconomic deprivation in the UK Biobank. *Am. J. Psychiatry* **178**, 625–634 (2021). 10.1176/appi.ajp.2020.20040462 33900812

[41] Conley D (2015). Is the effect of parental education on offspring biased or moderated by genotype?. Socio. Sci..

[42] Demange PA (2021). Investigating the genetic architecture of noncognitive skills using GWAS-by-subtraction. Nat. Genet..

[43] Borghans L, Golsteyn BHH, Heckman JJ, Humphries JE (2016). What grades and achievement tests measure. Proc. Natl Acad. Sci. USA.

[44] Bull R, Espy KA, Wiebe SA (2008). Short-term memory, working memory, and executive functioning in preschoolers: longitudinal predictors of mathematical achievement at age 7 years. Dev. Neuropsychol..

[45] Klingberg T (2010). Training and plasticity of working memory. Trends Cogn. Sci..

[46] Sauce B, Wiedenhoeft J, Judd N, Klingberg T (2021). Change by challenge: a common genetic basis behind childhood cognitive development and cognitive training. NPJ Sci. Learn.

[47] Garavan H (2018). Recruiting the ABCD sample: design considerations and procedures. Dev. Cogn. Neurosci..

[48] Strenze T (2007). Intelligence and socioeconomic success: a meta-analytic review of longitudinal research. Intelligence.

[49] Duncan, G. J. & Magnuson, K. A. *Socioeconomic Status, Parenting and Child Development* (Lawrence Erlbaum Associates, 2003).

[50] Reardon SF (2013). The widening income achievement gap. Educ. Leadersh..

[51] Selzam S (2019). Comparing within- and between-family polygenic score prediction. Am. J. Hum. Genet..

[52] Trejo S, Domingue BW (2018). Genetic nature or genetic nurture? Introducing social genetic parameters to quantify bias in polygenic score analyses. Biodemography Soc. Biol..

[53] Keller MC (2014). Gene × environment interaction studies have not properly controlled for potential confounders: the problem and the (simple) solution. Biol. Psychiatry.

[54] Kraft MA (2020). Interpreting effect sizes of education interventions. Educ. Res..

[55] Abelson RP (1985). A variance explanation paradox: when a little is a lot. Psychol. Bull..

[56] Pogrow S (2019). How effect size (Practical Significance) misleads clinical practice: the case for switching to practical benefit to assess applied research findings. Am. Stat..

[57] Mostafavi, H. et al. Variable prediction accuracy of polygenic scores within an ancestry group. *Elife* **9**, e48376 (2020). 10.7554/eLife.48376 PMC7067566 31999256

[58] Pain O (2021). Evaluation of polygenic prediction methodology within a reference-standardized framework. PLoS Genet..

[59] Anokhin AP (2022). Age-related changes and longitudinal stability of individual differences in ABCD neurocognition measures. Dev. Cogn. Neurosci..

[60] Schmitz L, Conley D (2017). Modeling gene-environment interactions with quasi-natural experiments. J. Pers..

[61] Dudbridge F, Fletcher O (2014). Gene-environment dependence creates spurious gene-environment interaction. Am. J. Hum. Genet..

[62] Wang, B. et al. Robust genetic nurture effects on education: a systematic review and meta-analysis based on 38,654 families across 8 cohorts. *Am. J. Hum. Genet*. 10.1016/j.ajhg.2021.07.010 (2021). 10.1016/j.ajhg.2021.07.010 PMC8456157 34416156

[63] Tucker-Drob, E. M. & Bates, T. C. Large cross-national differences in gene × socioeconomic status interaction on intelligence. *Psychol. Sci*. **27**, 138–149 (2016). 10.1177/0956797615612727 PMC4749462 26671911

[64] Figlio DN, Freese J, Karbownik K, Roth J (2017). Socioeconomic status and genetic influences on cognitive development. Proc. Natl Acad. Sci. USA.

[65] de Zeeuw EL (2019). The moderating role of SES on genetic differences in educational achievement in the Netherlands. NPJ Sci. Learn.

[66] Allegrini AG (2020). Multivariable G-E interplay in the prediction of educational achievement. PLoS Genet..

[67] Downey DB, Condron DJ (2016). Fifty years since the Coleman report: rethinking the relationship between schools and inequality. Sociol. Educ..

[68] Anvari, F. et al. x Not All Effects Are Indispensable: Psychological Science Requires Verifiable Lines of Reasoning for Whether an Effect Matters. *Perspect Psychol Sci: J Assoc Psychol Sci*, 10.1177/17456916221091565. (2022). 10.1177/17456916221091565 35994751

[69] Funder DC, Ozer DJ (2019). Evaluating effect size in psychological research: sense and nonsense. Adv. Methods Pract. Psychol. Sci..

[70] Fry A (2017). Comparison of sociodemographic and health-related characteristics of UK biobank participants with those of the general population. Am. J. Epidemiol..

[71] Domingue B, Trejo S, Armstrong-Carter E, Tucker-Drob E (2020). Interactions between polygenic scores and environments: methodological and conceptual challenges. Sociol. Sci..

[72] Becker J (2021). Resource profile and user guide of the polygenic index repository. Nat. Hum. Behav..

[73] Abdellaoui A, Verweij KJH (2021). Dissecting polygenic signals from genome-wide association studies on human behaviour. Nat. Hum. Behav..

[74] Luciana M (2018). Adolescent neurocognitive development and impacts of substance use: overview of the adolescent brain cognitive development (ABCD) baseline neurocognition battery. Dev. Cogn. Neurosci..

[75] Heeringa, S. G. & Berglund, P. A. A Guide for population-based analysis of the adolescent brain cognitive development (ABCD) study baseline data. Preprint at 10.1101/2020.02.10.942011 (2020).

[76] Heaton RK (2014). Reliability and validity of composite scores from the NIH Toolbox Cognition Battery in adults. J. Int. Neuropsychol. Soc..

[77] Gershon RC (2013). NIH Toolbox for assessment of neurological and behavioral function. Neurology.

[78] Kind AJH (2014). Neighborhood socioeconomic disadvantage and 30-day rehospitalization: a retrospective cohort study. Ann. Intern. Med..

[79] Baurley JW, Edlund CK, Pardamean CI, Conti DV, Bergen AW (2016). Smokescreen: a targeted genotyping array for addiction research. BMC Genomics.

[80] Howie, B. N., Donnelly, P. & Marchini, J. 1,000 genomes haplotypes—Phase 3 integrated variant set release in NCBI build 37 (hg19) coordinates. Preprint at (2015).

[81] 1000 Genomes Project Consortium. (2012). An integrated map of genetic variation from 1,092 human genomes. Nature.

[82] Choi, S. W. & O’Reilly, P. F. PRSice-2: polygenic risk score software for biobank-scale data. *Gigascience* **8**, giz082 (2019). 10.1093/gigascience/giz082 PMC6629542 31307061

[83] Turley P (2018). Multi-trait analysis of genome-wide association summary statistics using MTAG. Nat. Genet..

[84] Bates D, Mächler M, Bolker B, Walker S (2015). Fitting linear mixed-effects models using lme4. J. Stat. Softw..

[85] R Core Team. R: a language and environment for statistical computing. http://www.R-project.org/ (2014).

[86] Kuznetsova A, Brockhoff PB, Christensen RHB (2017). lmerTest package: tests in linear mixed effects models. J. Stat. Softw., Artic..

[87] Lundberg I, Johnson R, Stewart BM (2021). What is your estimand? Defining the target quantity connects statistical evidence to theory. Am. Sociol. Rev..

[88] Cliffordson C (2010). Methodological issues in investigations of the relative effects of schooling and age on school performance: the between-grade regression discontinuity design applied to Swedish TIMSS 1995 data. Educ. Res. Eval..

[89] Loughnan, R. J. et al. Gene-experience correlation during cognitive development: evidence from the adolescent brain cognitive development (ABCD) StudySM. Preprint at *bioRxiv*10.1101/637512 (2021).

[90] Hanscombe KB (2012). Socioeconomic status (SES) and children’s intelligence (IQ): in a UK-representative sample SES moderates the environmental, not genetic, effect on IQ. PLoS ONE.

[91] Plomin R, DeFries JC, Loehlin JC (1977). Genotype-environment interaction and correlation in the analysis of human behavior. Psychol. Bull..

[92] Rosseel Y (2012). Lavaan: an R package for structural equation modeling and more. Version 0.5–12 (BETA). J. Stat. Softw..

[93] Bürkner P-C (2017). brms: an R package for Bayesian multilevel models using Stan. J. Stat. Softw..

