# Supplementary material for: Schooling substantially improves intelligence, but neither lessens nor widens the impacts of socioeconomics and genetics
Source: NPJ Sci Learn. 2022 Dec 15;7:33. doi: 10.1038/s41539-022-00148-5 (PMC9755250; doi:10.1038/s41539-022-00148-5)
Supplement: Supplementary file 1 — Supplementary Material [file 41539_2022_148_MOESM1_ESM.pdf]

# Supplementary information

Schooling project ABCD
Nicholas Judd
01/09/2021
nickkjudd@gmail.com
github.com/njudd/ABCDschooling

# Supplementary Table 1

*Descriptive table of the full sample.*

| | n | mean | sd | median | trimmed | mad | min | max | range | skew | kurtosis | se |
|---|---|---|---|---|---|---|---|---|---|---|---|---|
| *c*IQ | 6567 | 0.00 | 1.00 | -0.01 | -0.01 | 0.91 | -2.46 | 2.44 | 4.90 | 0.10 | -0.08 | 0.01 |
| *f*IQ | 6567 | 0.00 | 1.00 | 0.07 | 0.02 | 1.02 | -2.77 | 2.72 | 5.49 | -0.23 | -0.02 | 0.01 |
| WM | 6567 | 0.00 | 1.00 | -0.06 | 0.03 | 1.05 | -2.45 | 2.34 | 4.79 | -0.29 | -0.20 | 0.01 |
| Age | 6567 | 0.00 | 1.00 | -0.08 | -0.02 | 1.22 | -1.59 | 1.85 | 3.44 | 0.10 | -1.21 | 0.01 |
| Age Unscaled | 6567 | 9.88 | 0.61 | 9.83 | 9.87 | 0.74 | 8.92 | 11.00 | 2.08 | 0.10 | -1.21 | 0.01 |
| Schooling | 6567 | 0.00 | 1.00 | 0.16 | -0.02 | 1.10 | -2.37 | 1.94 | 4.31 | 0.08 | -0.84 | 0.01 |
| Schooling Unscaled | 6567 | 1.69 | 0.67 | 1.80 | 1.68 | 0.74 | 0.10 | 3.00 | 2.90 | 0.08 | -0.84 | 0.01 |
| SES | 6567 | 0.00 | 1.00 | 0.14 | 0.04 | 0.97 | -2.88 | 3.19 | 6.07 | -0.32 | -0.19 | 0.01 |
| cogPGS | 6567 | 0.00 | 1.00 | 0.03 | 0.01 | 1.03 | -3.41 | 3.63 | 7.04 | -0.05 | -0.24 | 0.01 |
| Parental Income | 6093 | 0.00 | 1.00 | 0.26 | 0.14 | 0.64 | -2.75 | 1.12 | 3.87 | -1.15 | 0.57 | 0.01 |
| Max Parental Education | 6566 | 0.00 | 1.00 | 0.05 | 0.04 | 1.12 | -2.97 | 1.56 | 4.53 | -0.38 | -0.25 | 0.01 |
| Neighborhood Quality | 6304 | 0.00 | 1.00 | -0.23 | -0.17 | 0.61 | -1.35 | 3.63 | 4.99 | 1.95 | 4.13 | 0.01 |

# Supplementary Table 2

*Checks for multicollinearity using the full model for cIQ (i.e., Equation 4). Variance inflation factors (VIF) are all below 5, indicating low correlations. VIFs were almost identical across different dependent variables (i.e., cIQ, fIQ & WM).*

| **Term** | **VIF** | **Increased SE** |
|---|---|---|
| Age | 3.14 | 1.77 |
| Schooling | 3.37 | 1.84 |
| Sex | 1.01 | 1.00 |
| cogPGS | 1.45 | 1.21 |
| SES | 1.50 | 1.23 |
| C1 | 1.58 | 1.26 |
| C2 | 1.08 | 1.04 |
| C3 | 1.18 | 1.08 |
| C4 | 1.05 | 1.02 |
| C5 | 1.02 | 1.01 |
| C6 | 1.95 | 1.40 |
| C7 | 1.94 | 1.39 |
| C8 | 1.04 | 1.02 |
| C9 | 1.12 | 1.06 |
| C10 | 1.20 | 1.10 |
| C11 | 1.13 | 1.06 |
| C12 | 1.12 | 1.06 |
| C13 | 1.04 | 1.02 |
| C14 | 1.02 | 1.01 |
| C15 | 1.04 | 1.02 |
| C16 | 1.01 | 1.01 |
| C17 | 1.02 | 1.01 |
| C18 | 1.01 | 1.00 |
| C19 | 1.01 | 1.00 |
| C20 | 1.04 | 1.02 |
| cogPGS*SES | 1.11 | 1.05 |
| Schooling*cogPGS | 3.56 | 1.89 |
| Schooling*SES | 3.48 | 1.87 |
| Age*cogPGS | 3.51 | 1.87 |
| Age*SES | 3.39 | 1.84 |
| Schooling*cogPGS*SES | 1.23 | 1.11 |

# Supplementary Table 3

*Linear mixed-effects models for crystallized IQ (cIQ) including subjects for subjects with complete data (n =6567).*

| | ***c*IQ (Equation 1)** | | | ***c*IQ (Equation 2)** | | | ***c*IQ (Equation 3)** | | | ***c*IQ (Equation 4)** | | |
|---|---|---|---|---|---|---|---|---|---|---|---|---|
| *Predictors* | *Estimates* | *SE* | *p* | *Estimates* | *SE* | *p* | *Estimates* | *SE* | *p* | *Estimates* | *SE* | *p* |
| (Intercept) | 0.000 | 0.045 | 1.000 | -0.027 | 0.026 | 0.302 | -0.018 | 0.026 | 0.495 | -0.018 | 0.026 | 0.491 |
| Age | 0.144 | 0.021 | <0.001 | 0.129 | 0.018 | <0.001 | 0.131 | 0.018 | <0.001 | 0.132 | 0.018 | <0.001 |
| Schooling | 0.134 | 0.021 | <0.001 | 0.145 | 0.018 | <0.001 | 0.142 | 0.018 | <0.001 | 0.134 | 0.019 | <0.001 |
| Sex [M] | | | | 0.046 | 0.020 | 0.023 | 0.045 | 0.020 | 0.026 | 0.045 | 0.020 | 0.026 |
| cogPGS | | | | 0.159 | 0.012 | <0.001 | 0.161 | 0.012 | <0.001 | 0.161 | 0.012 | <0.001 |
| SES | | | | 0.291 | 0.013 | <0.001 | 0.288 | 0.013 | <0.001 | 0.289 | 0.013 | <0.001 |
| C1 | | | | -0.117 | 0.013 | <0.001 | -0.111 | 0.014 | <0.001 | -0.111 | 0.014 | <0.001 |
| C2 | | | | 0.057 | 0.012 | <0.001 | 0.059 | 0.012 | <0.001 | 0.059 | 0.012 | <0.001 |
| C3 | | | | 0.103 | 0.011 | <0.001 | 0.104 | 0.011 | <0.001 | 0.104 | 0.011 | <0.001 |
| C4 | | | | 0.014 | 0.011 | 0.187 | 0.014 | 0.011 | 0.194 | 0.014 | 0.011 | 0.195 |
| C5 | | | | 0.002 | 0.010 | 0.851 | 0.002 | 0.010 | 0.863 | 0.002 | 0.010 | 0.843 |
| C6 | | | | -0.003 | 0.014 | 0.846 | -0.004 | 0.014 | 0.796 | -0.004 | 0.014 | 0.792 |
| C7 | | | | 0.007 | 0.014 | 0.604 | 0.008 | 0.014 | 0.555 | 0.008 | 0.014 | 0.564 |
| C8 | | | | -0.022 | 0.011 | 0.040 | -0.023 | 0.011 | 0.035 | -0.022 | 0.011 | 0.040 |

| | | | | | | | | | |
|---|---|---|---|---|---|---|---|---|---|
| C9 | -0.037 | 0.011 | 0.001 | -0.037 | 0.011 | 0.001 | -0.037 | 0.011 | 0.001 |
| C10 | -0.011 | 0.011 | 0.347 | -0.011 | 0.011 | 0.316 | -0.011 | 0.011 | 0.320 |
| C11 | -0.034 | 0.011 | 0.002 | -0.035 | 0.011 | 0.002 | -0.035 | 0.011 | 0.001 |
| C12 | -0.011 | 0.011 | 0.324 | -0.012 | 0.011 | 0.269 | -0.012 | 0.011 | 0.262 |
| C13 | 0.001 | 0.010 | 0.947 | 0.000 | 0.010 | 0.979 | 0.000 | 0.010 | 0.970 |
| C14 | -0.008 | 0.010 | 0.435 | -0.007 | 0.010 | 0.479 | -0.008 | 0.010 | 0.463 |
| C15 | 0.005 | 0.010 | 0.620 | 0.005 | 0.010 | 0.616 | 0.005 | 0.010 | 0.614 |
| C16 | -0.007 | 0.010 | 0.521 | -0.007 | 0.010 | 0.512 | -0.007 | 0.010 | 0.505 |
| C17 | -0.001 | 0.010 | 0.914 | -0.002 | 0.010 | 0.881 | -0.001 | 0.010 | 0.908 |
| C18 | 0.010 | 0.010 | 0.333 | 0.010 | 0.010 | 0.347 | 0.009 | 0.010 | 0.351 |
| C19 | -0.008 | 0.010 | 0.433 | -0.008 | 0.010 | 0.416 | -0.008 | 0.010 | 0.428 |
| C20 | -0.020 | 0.010 | 0.051 | -0.020 | 0.010 | 0.052 | -0.021 | 0.010 | 0.047 |
| cogPGS * SES | | | | -0.020 | 0.011 | 0.063 | 0.020 | -0.011 | 0.066 |
| Schooling * cogPGS | | | | 0.003 | 0.019 | 0.877 | 0.004 | 0.019 | 0.828 |
| Schooling * SES | | | | -0.013 | 0.019 | 0.487 | -0.010 | 0.019 | 0.604 |
| Age * cogPGS | | | | 0.022 | 0.019 | 0.254 | 0.021 | 0.019 | 0.275 |
| Age * SES | | | | 0.026 | 0.019 | 0.176 | 0.025 | 0.019 | 0.189 |
| Schooling * cogPGS * SES | | | | | | | 0.018 | 0.010 | 0.092 |

| | | | | |
|---|---|---|---|---|
| **Random Effects** | | | | |
| $\sigma^2$ | 0.89 | 0.67 | 0.67 | 0.67 |
| $\tau_{00}$ | 0.04 $_{site\_id\_l}$ | 0.01 $_{site\_id\_l}$ | 0.01 $_{site\_id\_l}$ | 0.01 $_{site\_id\_l}$ |
| ICC | 0.04 | 0.01 | 0.01 | 0.01 |
| N | 22 $_{site\_id\_l}$ | 22 $_{site\_id\_l}$ | 22 $_{site\_id\_l}$ | 22 $_{site\_id\_l}$ |
| Observations | 6567 | 6567 | 6567 | 6567 |
| Marginal $R^2$ / Conditional $R^2$ | 0.070 / 0.112 | 0.330 / 0.340 | 0.331 / 0.341 | 0.332 / 0.341 |
| AIC | 17934.378 | 16104.673 | 16100.450 | 16099.616 |
| log-Likelihood | -8962.189 | -8024.337 | -8017.225 | -8015.808 |

# Supplementary Table 4

*Linear mixed-effects models for fluid IQ (fIQ) including subjects for subjects with complete data (n =6567).*

| | ***f*IQ (Equation 1)** | | | ***f*IQ (Equation 2)** | | | ***f*IQ (Equation 3)** | | | ***f*IQ (Equation 4)** | | |
|---|---|---|---|---|---|---|---|---|---|---|---|---|
| *Predictors* | *Estimates* | *SE* | *p* | *Estimates* | *SE* | *p* | *Estimates* | *SE* | *p* | *Estimates* | *SE* | *p* |
| (Intercept) | -0.009 | 0.044 | 0.845 | 0.041 | 0.024 | 0.088 | 0.045 | 0.025 | 0.070 | 0.045 | 0.025 | 0.070 |
| Age | 0.162 | 0.021 | <0.001 | 0.160 | 0.020 | <0.001 | 0.162 | 0.020 | <0.001 | 0.162 | 0.020 | <0.001 |
| Schooling | 0.100 | 0.021 | <0.001 | 0.096 | 0.020 | <0.001 | 0.095 | 0.020 | <0.001 | 0.097 | 0.020 | <0.001 |
| Sex [M] | | | | -0.094 | 0.022 | <0.001 | -0.095 | 0.022 | <0.001 | -0.095 | 0.022 | <0.001 |
| cogPGS | | | | 0.088 | 0.014 | <0.001 | 0.089 | 0.014 | <0.001 | 0.089 | 0.014 | <0.001 |
| SES | | | | 0.182 | 0.014 | <0.001 | 0.181 | 0.014 | <0.001 | 0.181 | 0.014 | <0.001 |
| C1 | | | | -0.131 | 0.014 | <0.001 | -0.130 | 0.015 | <0.001 | -0.130 | 0.015 | <0.001 |
| C2 | | | | -0.021 | 0.013 | 0.105 | -0.020 | 0.013 | 0.121 | -0.020 | 0.013 | 0.121 |
| C3 | | | | 0.079 | 0.012 | <0.001 | 0.080 | 0.012 | <0.001 | 0.080 | 0.012 | <0.001 |
| C4 | | | | 0.016 | 0.012 | 0.168 | 0.016 | 0.012 | 0.178 | 0.016 | 0.012 | 0.178 |
| C5 | | | | 0.003 | 0.011 | 0.818 | 0.003 | 0.011 | 0.812 | 0.003 | 0.011 | 0.818 |
| C6 | | | | 0.009 | 0.015 | 0.573 | 0.008 | 0.015 | 0.586 | 0.008 | 0.015 | 0.585 |
| C7 | | | | 0.053 | 0.015 | 0.001 | 0.054 | 0.015 | <0.001 | 0.054 | 0.015 | <0.001 |

| | | | | | | | | | |
|---|---|---|---|---|---|---|---|---|---|
| C8 | -0.020 | 0.012 | 0.087 | -0.021 | 0.012 | 0.078 | -0.021 | 0.012 | 0.075 |
| C9 | 0.009 | 0.012 | 0.471 | 0.009 | 0.012 | 0.467 | 0.009 | 0.012 | 0.466 |
| C10 | 0.009 | 0.012 | 0.438 | 0.009 | 0.012 | 0.439 | 0.009 | 0.012 | 0.440 |
| C11 | -0.017 | 0.012 | 0.164 | -0.017 | 0.012 | 0.153 | -0.017 | 0.012 | 0.155 |
| C12 | -0.008 | 0.012 | 0.490 | -0.008 | 0.012 | 0.480 | -0.008 | 0.012 | 0.483 |
| C13 | -0.006 | 0.011 | 0.589 | -0.006 | 0.011 | 0.586 | -0.006 | 0.011 | 0.584 |
| C14 | -0.013 | 0.011 | 0.247 | -0.013 | 0.011 | 0.253 | -0.013 | 0.011 | 0.257 |
| C15 | 0.017 | 0.011 | 0.135 | 0.017 | 0.011 | 0.141 | 0.017 | 0.011 | 0.141 |
| C16 | -0.002 | 0.011 | 0.857 | -0.002 | 0.011 | 0.872 | -0.002 | 0.011 | 0.874 |
| C17 | 0.012 | 0.011 | 0.301 | 0.012 | 0.011 | 0.296 | 0.012 | 0.011 | 0.301 |
| C18 | 0.005 | 0.011 | 0.680 | 0.005 | 0.011 | 0.642 | 0.005 | 0.011 | 0.640 |
| C19 | -0.005 | 0.011 | 0.671 | -0.005 | 0.011 | 0.666 | -0.005 | 0.011 | 0.662 |
| C20 | -0.007 | 0.011 | 0.523 | -0.007 | 0.011 | 0.530 | -0.007 | 0.011 | 0.539 |
| cogPGS * SES | | | | -0.006 | 0.012 | 0.621 | -0.006 | 0.012 | 0.618 |
| Schooling * cogPGS | | | | -0.019 | 0.021 | 0.362 | -0.019 | 0.021 | 0.353 |
| Schooling * SES | | | | -0.013 | 0.020 | 0.511 | -0.014 | 0.020 | 0.480 |
| Age * cogPGS | | | | 0.015 | 0.021 | 0.479 | 0.015 | 0.021 | 0.470 |
| Age * SES | | | | 0.007 | 0.021 | 0.746 | 0.007 | 0.021 | 0.738 |

| | | | | | | | | | | | | |
|---|---|---|---|---|---|---|---|---|---|---|---|---|
| Schooling * cogPGS * SES | | | | | | | | | | -0.006 | 0.011 | 0.608 |
| **Random Effects** | | | | | | | | | | | | |
| $\sigma^2$ | 0.90 | | | 0.80 | | | 0.80 | | | 0.80 | | |
| $\tau_{00}$ | 0.04 $_{site\_id\_1}$ | | | 0.01 $_{site\_id\_1}$ | | | 0.01 $_{site\_id\_1}$ | | | 0.01 $_{site\_id\_1}$ | | |
| ICC | 0.04 | | | 0.01 | | | 0.01 | | | 0.01 | | |
| N | 22 $_{site\_id\_1}$ | | | 22 $_{site\_id\_1}$ | | | 22 $_{site\_id\_1}$ | | | 22 $_{site\_id\_1}$ | | |
| Observations | 6567 | | | 6567 | | | 6567 | | | 6567 | | |
| Marginal $R^2$ / Conditional $R^2$ | 0.062 / 0.101 | | | 0.195 / 0.202 | | | 0.195 / 0.202 | | | 0.195 / 0.202 | | |
| AIC | 18017.919 | | | 17218.323 | | | 17225.680 | | | 17227.416 | | |
| log-Likelihood | -9003.960 | | | -8581.162 | | | -8579.840 | | | -8579.708 | | |

# Supplementary Table 5

*Linear mixed-effects models for Working Memory list sorting (WM) including subjects for subjects with complete data (n =6567).*

| | **WM (Equation 1)** | | | **WM (Equation 2)** | | | **WM (Equation 3)** | | | **WM (Equation 4)** | | |
|---|---|---|---|---|---|---|---|---|---|---|---|---|
| *Predictors* | *Estimates* | *SE* | *p* | *Estimates* | *SE* | *p* | *Estimates* | *SE* | *p* | *Estimates* | *SE* | *p* |
| (Intercept) | -0.009 | 0.034 | 0.796 | -0.046 | 0.022 | 0.036 | -0.047 | 0.023 | 0.038 | -0.047 | 0.023 | 0.038 |
| Age | 0.052 | 0.021 | 0.016 | 0.039 | 0.020 | 0.055 | 0.039 | 0.020 | 0.057 | 0.039 | 0.020 | 0.058 |
| Schooling | 0.087 | 0.021 | <0.001 | 0.095 | 0.020 | <0.001 | 0.095 | 0.020 | <0.001 | 0.096 | 0.021 | <0.001 |
| Sex [M] | | | | 0.078 | 0.023 | 0.001 | 0.078 | 0.023 | 0.001 | 0.078 | 0.023 | 0.001 |
| cogPGS | | | | 0.086 | 0.014 | <0.001 | 0.086 | 0.014 | <0.001 | 0.086 | 0.014 | <0.001 |
| SES | | | | 0.215 | 0.015 | <0.001 | 0.215 | 0.015 | <0.001 | 0.215 | 0.015 | <0.001 |
| C1 | | | | -0.111 | 0.015 | <0.001 | -0.111 | 0.015 | <0.001 | -0.111 | 0.015 | <0.001 |
| C2 | | | | 0.023 | 0.013 | 0.075 | 0.022 | 0.013 | 0.082 | 0.022 | 0.013 | 0.082 |
| C3 | | | | 0.041 | 0.013 | 0.001 | 0.041 | 0.013 | 0.001 | 0.041 | 0.013 | 0.001 |
| C4 | | | | 0.023 | 0.012 | 0.060 | 0.022 | 0.012 | 0.064 | 0.022 | 0.012 | 0.064 |
| C5 | | | | 0.002 | 0.012 | 0.872 | 0.002 | 0.012 | 0.868 | 0.002 | 0.012 | 0.869 |
| C6 | | | | 0.020 | 0.016 | 0.214 | 0.020 | 0.016 | 0.211 | 0.020 | 0.016 | 0.211 |
| C7 | | | | 0.052 | 0.016 | 0.001 | 0.052 | 0.016 | 0.001 | 0.052 | 0.016 | 0.001 |
| C8 | | | | -0.011 | 0.012 | 0.342 | -0.011 | 0.012 | 0.347 | -0.011 | 0.012 | 0.346 |

| | | | | | | | | | |
|---|---|---|---|---|---|---|---|---|---|
| C9 | -0.002 | 0.012 | 0.848 | -0.002 | 0.012 | 0.860 | -0.002 | 0.012 | 0.860 |
| C10 | -0.004 | 0.013 | 0.779 | -0.003 | 0.013 | 0.783 | -0.003 | 0.013 | 0.783 |
| C11 | -0.023 | 0.012 | 0.059 | -0.023 | 0.012 | 0.057 | -0.023 | 0.012 | 0.057 |
| C12 | 0.000 | 0.012 | 0.980 | 0.000 | 0.012 | 0.977 | 0.000 | 0.012 | 0.976 |
| C13 | -0.003 | 0.012 | 0.819 | -0.003 | 0.012 | 0.811 | -0.003 | 0.012 | 0.811 |
| C14 | -0.008 | 0.012 | 0.471 | -0.008 | 0.012 | 0.464 | -0.008 | 0.012 | 0.465 |
| C15 | 0.006 | 0.012 | 0.615 | 0.006 | 0.012 | 0.624 | 0.006 | 0.012 | 0.624 |
| C16 | 0.003 | 0.011 | 0.760 | 0.004 | 0.011 | 0.759 | 0.004 | 0.011 | 0.759 |
| C17 | -0.004 | 0.012 | 0.740 | -0.004 | 0.012 | 0.739 | -0.004 | 0.012 | 0.738 |
| C18 | 0.018 | 0.011 | 0.113 | 0.019 | 0.011 | 0.104 | 0.019 | 0.011 | 0.104 |
| C19 | -0.006 | 0.011 | 0.600 | -0.006 | 0.011 | 0.604 | -0.006 | 0.011 | 0.603 |
| C20 | 0.005 | 0.012 | 0.657 | 0.005 | 0.012 | 0.665 | 0.005 | 0.012 | 0.663 |
| cogPGS * SES | | | | -0.002 | 0.012 | 0.887 | -0.002 | 0.012 | 0.888 |
| Schooling * cogPGS | | | | -0.021 | 0.022 | 0.335 | -0.021 | 0.022 | 0.334 |
| Schooling * SES | | | | -0.012 | 0.021 | 0.561 | -0.012 | 0.021 | 0.570 |
| Age * cogPGS | | | | 0.007 | 0.021 | 0.756 | 0.007 | 0.021 | 0.754 |
| Age * SES | | | | 0.003 | 0.021 | 0.892 | 0.003 | 0.021 | 0.894 |
| Schooling * cogPGS * SES | | | | | | | 0.001 | 0.012 | 0.929 |

| **Random Effects** | | | | |
|---|---|---|---|---|
| $\sigma^2$ | 0.96 | 0.85 | 0.85 | 0.85 |
| $\tau_{00}$ | 0.02 $_{site\_id\_1}$ | 0.00 $_{site\_id\_1}$ | 0.00 $_{site\_id\_1}$ | 0.00 $_{site\_id\_1}$ |
| ICC | 0.02 | 0.01 | 0.01 | 0.01 |
| N | 22 $_{site\_id\_1}$ | 22 $_{site\_id\_1}$ | 22 $_{site\_id\_1}$ | 22 $_{site\_id\_1}$ |
| Observations | 6567 | 6567 | 6567 | 6567 |
| Marginal $R^2$ / Conditional $R^2$ | 0.018 / 0.039 | 0.147 / 0.152 | 0.148 / 0.152 | 0.148 / 0.152 |
| AIC | 18419.937 | 17650.254 | 17658.583 | 17660.575 |
| log-Likelihood | -9204.968 | -8797.127 | -8796.291 | -8796.287 |

# Supplementary Table 6

*Post hoc Linear mixed-effects models for g including subjects for subjects with complete data (n =6567). P-values are FDR corrected.*

| | **g (Equation 1)** | | | **g (Equation 2)** | | | **g (Equation 3)** | | | **g (Equation 4)** | | |
|---|---|---|---|---|---|---|---|---|---|---|---|---|
| *Predictors* | *Estimates* | *SE* | *p* | *Estimates* | *SE* | *p* | *Estimates* | *SE* | *p* | *Estimates* | *SE* | *p* |
| (Intercept) | -0.007 | 0.049 | 0.895 | -0.004 | 0.025 | 0.921 | 0.003 | 0.025 | 0.976 | 0.003 | 0.025 | 0.999 |
| Age | 0.146 | 0.021 | <0.001 | 0.136 | 0.018 | <0.001 | 0.137 | 0.018 | <0.001 | 0.137 | 0.018 | <0.001 |
| Schooling | 0.129 | 0.021 | <0.001 | 0.134 | 0.018 | <0.001 | 0.134 | 0.018 | <0.001 | 0.133 | 0.019 | <0.001 |
| Sex [M] | | | | -0.009 | 0.020 | 0.792 | -0.010 | 0.020 | 0.829 | -0.010 | 0.020 | 0.855 |
| cogPGS | | | | 0.145 | 0.012 | <0.001 | 0.146 | 0.012 | <0.001 | 0.146 | 0.012 | <0.001 |
| SES | | | | 0.301 | 0.013 | <0.001 | 0.299 | 0.013 | <0.001 | 0.299 | 0.013 | <0.001 |
| C1 | | | | -0.146 | 0.013 | <0.001 | -0.142 | 0.013 | <0.001 | -0.142 | 0.013 | <0.001 |
| C2 | | | | 0.022 | 0.012 | 0.150 | 0.023 | 0.012 | 0.146 | 0.023 | 0.012 | 0.151 |
| C3 | | | | 0.086 | 0.011 | <0.001 | 0.087 | 0.011 | <0.001 | 0.087 | 0.011 | <0.001 |
| C4 | | | | 0.018 | 0.011 | 0.195 | 0.018 | 0.011 | 0.241 | 0.018 | 0.011 | 0.249 |
| C5 | | | | 0.007 | 0.010 | 0.706 | 0.007 | 0.010 | 0.799 | 0.007 | 0.010 | 0.825 |
| C6 | | | | 0.003 | 0.014 | 0.915 | 0.002 | 0.014 | 0.976 | 0.002 | 0.014 | 0.999 |
| C7 | | | | 0.038 | 0.014 | 0.024 | 0.039 | 0.014 | 0.024 | 0.039 | 0.014 | 0.025 |
| C8 | | | | -0.023 | 0.011 | 0.079 | -0.025 | 0.011 | 0.074 | -0.025 | 0.011 | 0.077 |

| | | | | | | | | | |
|---|---|---|---|---|---|---|---|---|---|
| C9 | -0.006 | 0.011 | 0.789 | -0.006 | 0.011 | 0.823 | -0.006 | 0.011 | 0.849 |
| C10 | 0.005 | 0.011 | 0.792 | 0.005 | 0.011 | 0.829 | 0.005 | 0.011 | 0.855 |
| C11 | -0.027 | 0.011 | 0.049 | -0.027 | 0.011 | 0.052 | -0.027 | 0.011 | 0.054 |
| C12 | -0.009 | 0.011 | 0.635 | -0.010 | 0.011 | 0.662 | -0.010 | 0.011 | 0.683 |
| C13 | -0.003 | 0.010 | 0.882 | -0.004 | 0.010 | 0.907 | -0.004 | 0.010 | 0.936 |
| C14 | -0.013 | 0.010 | 0.397 | -0.013 | 0.010 | 0.460 | -0.013 | 0.010 | 0.475 |
| C15 | 0.012 | 0.010 | 0.431 | 0.012 | 0.010 | 0.498 | 0.012 | 0.010 | 0.514 |
| C16 | 0.001 | 0.010 | 0.940 | 0.001 | 0.010 | 0.976 | 0.001 | 0.010 | 0.999 |
| C17 | 0.003 | 0.010 | 0.882 | 0.003 | 0.010 | 0.941 | 0.003 | 0.010 | 0.971 |
| C18 | 0.018 | 0.010 | 0.165 | 0.019 | 0.010 | 0.185 | 0.019 | 0.010 | 0.191 |
| C19 | -0.007 | 0.010 | 0.706 | -0.007 | 0.010 | 0.791 | -0.007 | 0.010 | 0.817 |
| C20 | -0.010 | 0.010 | 0.545 | -0.010 | 0.010 | 0.600 | -0.010 | 0.010 | 0.619 |
| cogPGS * SES | | | | -0.015 | 0.011 | 0.378 | -0.015 | 0.011 | 0.390 |
| Schooling * cogPGS | | | | -0.009 | 0.019 | 0.829 | -0.009 | 0.019 | 0.855 |
| Schooling * SES | | | | -0.000 | 0.018 | 0.998 | 0.000 | 0.019 | 0.999 |
| Age * cogPGS | | | | 0.011 | 0.019 | 0.823 | 0.011 | 0.019 | 0.849 |
| Age * SES | | | | 0.001 | 0.019 | 0.976 | 0.001 | 0.019 | 0.999 |
| Schooling * cogPGS * SES | | | | | | | 0.000 | 0.010 | 0.999 |

| | | | | |
|---|---|---|---|---|
| **Random Effects** | | | | |
| $\sigma^2$ | 0.88 | 0.66 | 0.66 | 0.66 |
| $\tau_{00}$ | 0.05 $_{site\_id\_1}$ | 0.01 $_{site\_id\_1}$ | 0.01 $_{site\_id\_1}$ | 0.01 $_{site\_id\_1}$ |
| ICC | 0.05 | 0.01 | 0.01 | 0.01 |
| N | 22 $_{site\_id\_1}$ | 22 $_{site\_id\_1}$ | 22 $_{site\_id\_1}$ | 22 $_{site\_id\_1}$ |
| Observations | 6567 | 6567 | 6567 | 6567 |
| Marginal $R^2$ / Conditional $R^2$ | 0.069 / 0.119 | 0.337 / 0.345 | 0.337 / 0.346 | 0.337 / 0.346 |
| AIC | 17890.653 | 16004.406 | 16011.960 | 16013.959 |
| log-Likelihood | -8940.326 | -7974.203 | -7972.980 | -7972.979 |

## Supplementary Table 7

*Post hoc Linear mixed-effects models of Equation 4 for each cognitive variable including European ancestry subjects defined with 4-means clustering on the first two principal components (n =3715). P-values are FDR corrected and variables are restandardized on the population.*

| | **cIQ (Equation 4)** | | | **fIQ (Equation 4)** | | | **WM (Equation 4)** | | | **g (Equation 4)** | | |
|---|---|---|---|---|---|---|---|---|---|---|---|---|
| *Predictors* | *Estimates* | *SE* | *p* | *Estimates* | *SE* | *p* | *Estimates* | *SE* | *p* | *Estimates* | *SE* | *p* |
| (Intercept) | -0.031 | 0.035 | 0.621 | 0.039 | 0.030 | 0.531 | -0.065 | 0.028 | 0.134 | -0.016 | 0.033 | 0.805 |
| Age | 0.148 | 0.027 | <0.001 | 0.177 | 0.029 | <0.001 | 0.031 | 0.030 | 0.804 | 0.145 | 0.027 | <0.001 |
| Schooling | 0.172 | 0.028 | <0.001 | 0.103 | 0.029 | 0.003 | 0.113 | 0.030 | 0.002 | 0.172 | 0.028 | <0.001 |
| Sex [M] | 0.071 | 0.030 | 0.087 | -0.112 | 0.031 | 0.003 | 0.100 | 0.032 | 0.014 | 0.006 | 0.030 | 0.885 |
| cogPGS | 0.170 | 0.015 | <0.001 | 0.094 | 0.016 | <0.001 | 0.106 | 0.016 | <0.001 | 0.166 | 0.015 | <0.001 |
| SES | 0.208 | 0.016 | <0.001 | 0.135 | 0.017 | <0.001 | 0.158 | 0.017 | <0.001 | 0.228 | 0.016 | <0.001 |
| C1 | -0.042 | 0.026 | 0.383 | 0.019 | 0.027 | 0.761 | -0.017 | 0.028 | 0.851 | -0.017 | 0.026 | 0.805 |
| C2 | -0.030 | 0.019 | 0.383 | -0.010 | 0.020 | 0.801 | -0.014 | 0.020 | 0.851 | -0.032 | 0.019 | 0.535 |
| C3 | 0.018 | 0.017 | 0.588 | 0.022 | 0.018 | 0.531 | 0.007 | 0.019 | 0.851 | 0.018 | 0.017 | 0.781 |
| C4 | 0.008 | 0.028 | 0.856 | 0.042 | 0.030 | 0.531 | 0.016 | 0.030 | 0.851 | 0.019 | 0.028 | 0.805 |
| C5 | 0.013 | 0.018 | 0.653 | 0.014 | 0.019 | 0.760 | 0.002 | 0.020 | 0.928 | 0.011 | 0.018 | 0.805 |
| C6 | -0.010 | 0.015 | 0.666 | -0.008 | 0.016 | 0.801 | 0.007 | 0.017 | 0.851 | -0.003 | 0.015 | 0.885 |
| C7 | 0.002 | 0.017 | 0.894 | 0.032 | 0.018 | 0.374 | 0.026 | 0.018 | 0.711 | 0.008 | 0.017 | 0.805 |

| | | | | | | | | | | | | |
|---|---|---|---|---|---|---|---|---|---|---|---|---|
| C8 | -0.031 | 0.020 | 0.383 | -0.007 | 0.021 | 0.803 | -0.015 | 0.021 | 0.851 | -0.029 | 0.020 | 0.640 |
| C9 | -0.051 | 0.017 | 0.015 | 0.006 | 0.018 | 0.803 | -0.006 | 0.018 | 0.851 | -0.014 | 0.017 | 0.805 |
| C10 | -0.014 | 0.017 | 0.621 | 0.008 | 0.018 | 0.803 | -0.022 | 0.018 | 0.804 | -0.004 | 0.017 | 0.885 |
| C11 | -0.024 | 0.017 | 0.422 | -0.014 | 0.018 | 0.760 | 0.006 | 0.018 | 0.851 | -0.007 | 0.017 | 0.812 |
| C12 | -0.010 | 0.016 | 0.666 | -0.014 | 0.017 | 0.760 | 0.009 | 0.017 | 0.851 | -0.007 | 0.016 | 0.805 |
| C13 | 0.009 | 0.016 | 0.670 | 0.010 | 0.017 | 0.801 | 0.017 | 0.017 | 0.804 | 0.020 | 0.016 | 0.753 |
| C14 | -0.016 | 0.015 | 0.588 | -0.022 | 0.016 | 0.531 | 0.003 | 0.016 | 0.909 | -0.017 | 0.015 | 0.763 |
| C15 | 0.011 | 0.015 | 0.653 | 0.026 | 0.016 | 0.495 | 0.016 | 0.017 | 0.804 | 0.022 | 0.015 | 0.640 |
| C16 | -0.012 | 0.015 | 0.637 | -0.006 | 0.016 | 0.803 | -0.009 | 0.016 | 0.851 | -0.008 | 0.015 | 0.805 |
| C17 | -0.003 | 0.015 | 0.888 | 0.018 | 0.016 | 0.531 | -0.002 | 0.016 | 0.909 | 0.001 | 0.015 | 0.950 |
| C18 | 0.008 | 0.015 | 0.670 | 0.002 | 0.015 | 0.944 | 0.023 | 0.016 | 0.711 | 0.021 | 0.015 | 0.640 |
| C19 | -0.013 | 0.015 | 0.621 | -0.000 | 0.016 | 0.985 | -0.005 | 0.016 | 0.851 | -0.003 | 0.015 | 0.885 |
| C20 | -0.020 | 0.015 | 0.422 | -0.018 | 0.016 | 0.531 | 0.006 | 0.017 | 0.851 | -0.015 | 0.015 | 0.781 |
| cogPGS * SES | -0.027 | 0.015 | 0.306 | 0.018 | 0.016 | 0.531 | 0.019 | 0.016 | 0.804 | -0.007 | 0.015 | 0.805 |
| Schooling * cogPGS | -0.005 | 0.027 | 0.888 | -0.014 | 0.029 | 0.801 | -0.014 | 0.029 | 0.851 | -0.014 | 0.027 | 0.805 |
| Schooling * SES | -0.026 | 0.027 | 0.621 | -0.044 | 0.028 | 0.495 | -0.004 | 0.029 | 0.909 | -0.030 | 0.027 | 0.763 |
| Age * cogPGS | 0.037 | 0.027 | 0.422 | 0.020 | 0.028 | 0.761 | 0.023 | 0.029 | 0.851 | 0.026 | 0.027 | 0.781 |
| Age * SES | 0.023 | 0.027 | 0.622 | 0.036 | 0.029 | 0.531 | 0.013 | 0.029 | 0.851 | 0.021 | 0.027 | 0.805 |

| | | | | | | | | | | | | |
|---|---|---|---|---|---|---|---|---|---|---|---|---|
| Schooling * cogPGS * SES | 0.019 | 0.014 | 0.422 | -0.005 | 0.015 | 0.803 | 0.016 | 0.015 | 0.804 | 0.009 | 0.014 | 0.805 |
| **Random Effects** | | | | | | | | | | | | |
| $\sigma^2$ | 0.79 | | | 0.88 | | | 0.93 | | | 0.79 | | |
| $\tau_{00}$ | 0.01 site_id_l | | | 0.01 site_id_l | | | 0.00 site_id_l | | | 0.01 site_id_l | | |
| ICC | 0.02 | | | 0.01 | | | 0.00 | | | 0.01 | | |
| N | 22 site_id_l | | | 22 site_id_l | | | 22 site_id_l | | | 22 site_id_l | | |
| Observations | 3715 | | | 3715 | | | 3715 | | | 3715 | | |
| Marginal $R^2$ / Conditional $R^2$ | 0.203 / 0.217 | | | 0.117 / 0.124 | | | 0.072 / 0.077 | | | 0.203 / 0.214 | | |
| AIC | 9778.428 | | | 10141.305 | | | 10337.290 | | | 9775.487 | | |
| log-Likelihood | -4855.214 | | | -5036.653 | | | -5134.645 | | | -4853.744 | | |

# Supplementary Table 8

*Region of practical equivalence (ROPE) boundary table from a Bayesian mixed effects model of Equation 3 with boundaries .05 SD and .02 SD on 95% highest density intervals (HDI). Values indicate the percentage of the 95 HDI within the ROPE boundary. See methods for more information on weakly informative priors (results were similar with the default flat priors). The 3-way interaction term comes from Equation 4 with an addition prior with a mean of 0 and an SD of .1.*

| **Parameter** | **HDI** | **cIQ ROPE (.05 sd)** | **fIQ ROPE (.05 sd)** | **WM ROPE (.05 sd)** | **cIQ ROPE (.02 sd)** | **fIQ ROPE (.02 sd)** | **WM ROPE (.02 sd)** |
|---|---|---|---|---|---|---|---|
| cogPGS * SES | 0.95 | 100% | 100% | 100% | 51% | 91% | 94% |
| Schooling * cogPGS | 0.95 | 100% | 97% | 95% | 75% | 53% | 50% |
| Schooling * SES | 0.95 | 100% | 100% | 100% | 67% | 63% | 64% |
| Schooling * cogPGS * SES | 0.95 | 100% | 100% | 100% | 61% | 92% | 96% |

# Supplementary Table 9a

*Recruitment was throughout the year in ABCD, this makes controlling for the month of schooling a better option that grade (as there is within-grade months of schooling variance). We carried out two supplementary sensitivity analyses where we recoded schooling a) coding grade as ordinal (i.e., 3, 4, 5) and another b) where we excluded the grade with the least subjects (3rd grade) and added a dummy coded categorical variable for fifth grade. P-values are uncorrected.*

| | ***c*IQ ordinal coding** | | | ***f*IQ ordinal coding** | | | **WM ordinal coding** | | |
|---|---|---|---|---|---|---|---|---|---|
| *Predictors* | *Estimates* | *SE* | *p* | *Estimates* | *SE* | *p* | *Estimates* | *SE* | *p* |
| (Intercept) | -3.453 | 0.237 | <0.001 | -3.616 | 0.239 | <0.001 | -1.504 | 0.244 | <0.001 |
| Age Unscaled | 0.288 | 0.031 | <0.001 | 0.333 | 0.032 | <0.001 | 0.106 | 0.033 | 0.001 |
| Grade Ordinal | 0.146 | 0.027 | <0.001 | 0.077 | 0.027 | 0.005 | 0.106 | 0.028 | <0.001 |
| **Random Effects** | | | | | | | | | |
| $\sigma^2$ | 0.89 | | | 0.90 | | | 0.96 | | |
| $\tau_{00}$ | 0.04 $_{site\_id\_l}$ | | | 0.04 $_{site\_id\_l}$ | | | 0.02 $_{site\_id\_l}$ | | |
| ICC | 0.05 | | | 0.04 | | | 0.02 | | |
| N | 22 $_{site\_id\_l}$ | | | 22 $_{site\_id\_l}$ | | | 22 $_{site\_id\_l}$ | | |
| Observations | 6567 | | | 6567 | | | 6567 | | |
| Marginal $R^2$ / Conditional $R^2$ | 0.068 / 0.111 | | | 0.060 / 0.100 | | | 0.017 / 0.039 | | |
| AIC | 17947.668 | | | 18032.967 | | | 18421.990 | | |
| log-Likelihood | -8968.834 | | | -9011.484 | | | -9205.995 | | |

## Supplementary Table 9b

*Recruitment was throughout the year in ABCD, this makes controlling for the month of schooling a better option that grade (as there is within-grade months of schooling variance). We carried out two supplementary sensitivity analyses where we recoded schooling a) coding grade as ordinal (i.e., 3, 4, 5) and another b) where we excluded the grade with the least subjects (3rd grade) and added a dummy coded categorical variable for fifth grade. P-values are uncorrected.*

| | **cIQ dummy coding** | | | **fIQ dummy coding** | | | **WM dummy coding** | | |
|---|---|---|---|---|---|---|---|---|---|
| *Predictors* | *Estimates* | *std. Error* | *p* | *Estimates* | *std. Error* | *p* | *Estimates* | *std. Error* | *p* |
| (Intercept) | -2.926 | 0.327 | <0.001 | -3.374 | 0.325 | <0.001 | -1.152 | 0.332 | 0.001 |
| Age Unscaled | 0.293 | 0.033 | <0.001 | 0.338 | 0.033 | <0.001 | 0.113 | 0.034 | 0.001 |
| 5th grade TRUE | 0.159 | 0.037 | <0.001 | 0.092 | 0.037 | 0.014 | 0.114 | 0.038 | 0.003 |
| **Random Effects** | | | | | | | | | |
| $\sigma^2$ | 0.91 | | | 0.90 | | | 0.95 | | |
| $\tau_{00}$ | 0.04 $_{site\_id\_l}$ | | | 0.03 $_{site\_id\_l}$ | | | 0.02 $_{site\_id\_l}$ | | |
| ICC | 0.04 | | | 0.04 | | | 0.02 | | |
| N | 22 $_{site\_id\_l}$ | | | 22 $_{site\_id\_l}$ | | | 22 $_{site\_id\_l}$ | | |
| Observations | 5458 | | | 5458 | | | 5458 | | |
| Marginal $R^2$ / Conditional $R^2$ | 0.051 / 0.091 | | | 0.051 / 0.085 | | | 0.012 / 0.031 | | |
| AIC | 15023.598 | | | 14970.674 | | | 15245.739 | | |
| log-Likelihood | -7506.799 | | | -7480.337 | | | -7617.869 | | |

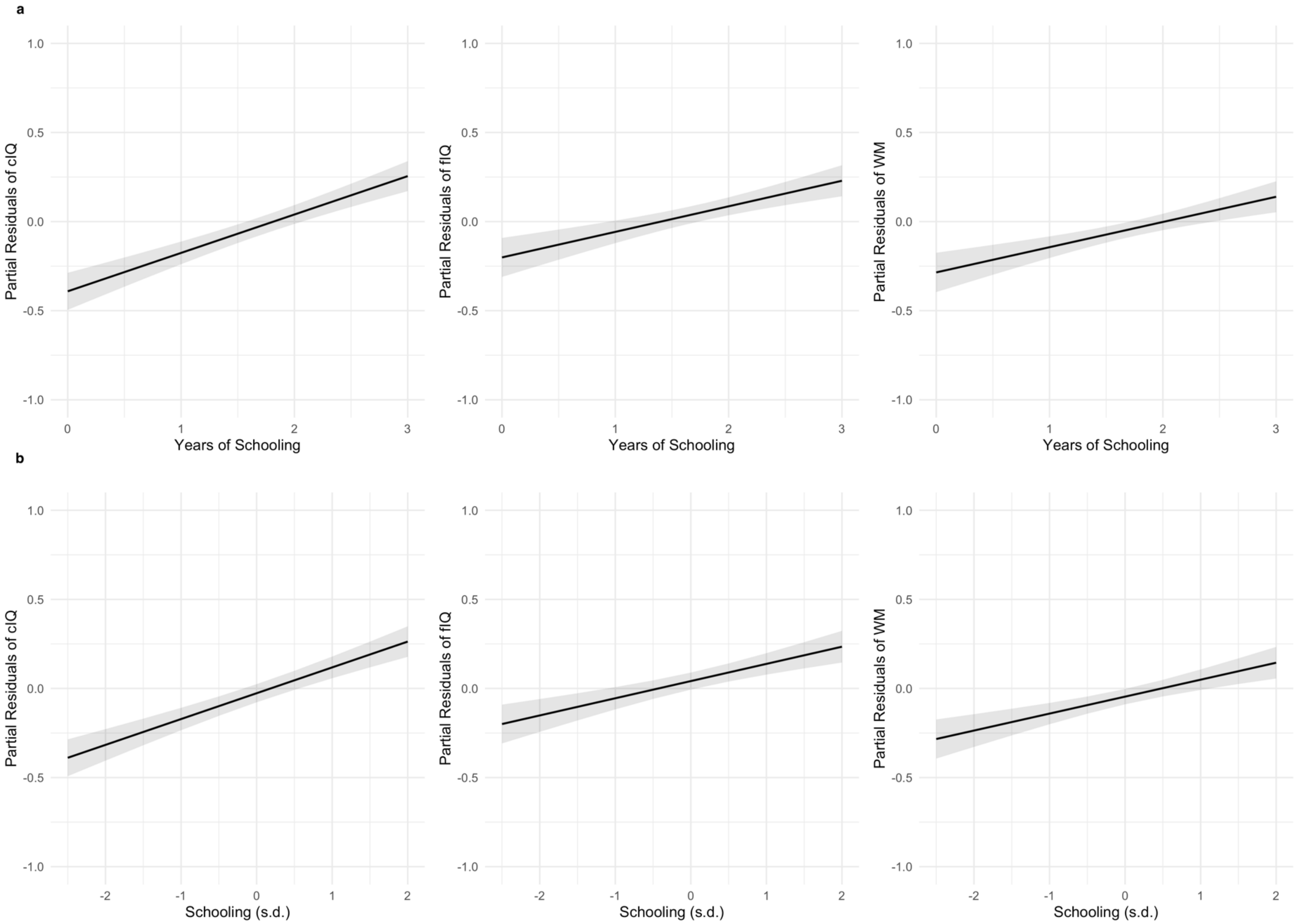


*Supplementary Figure 1:* A partial residual plot for a) years of schooling and b) Schooling in standard units for *c*IQ, *f*IQ and WM.

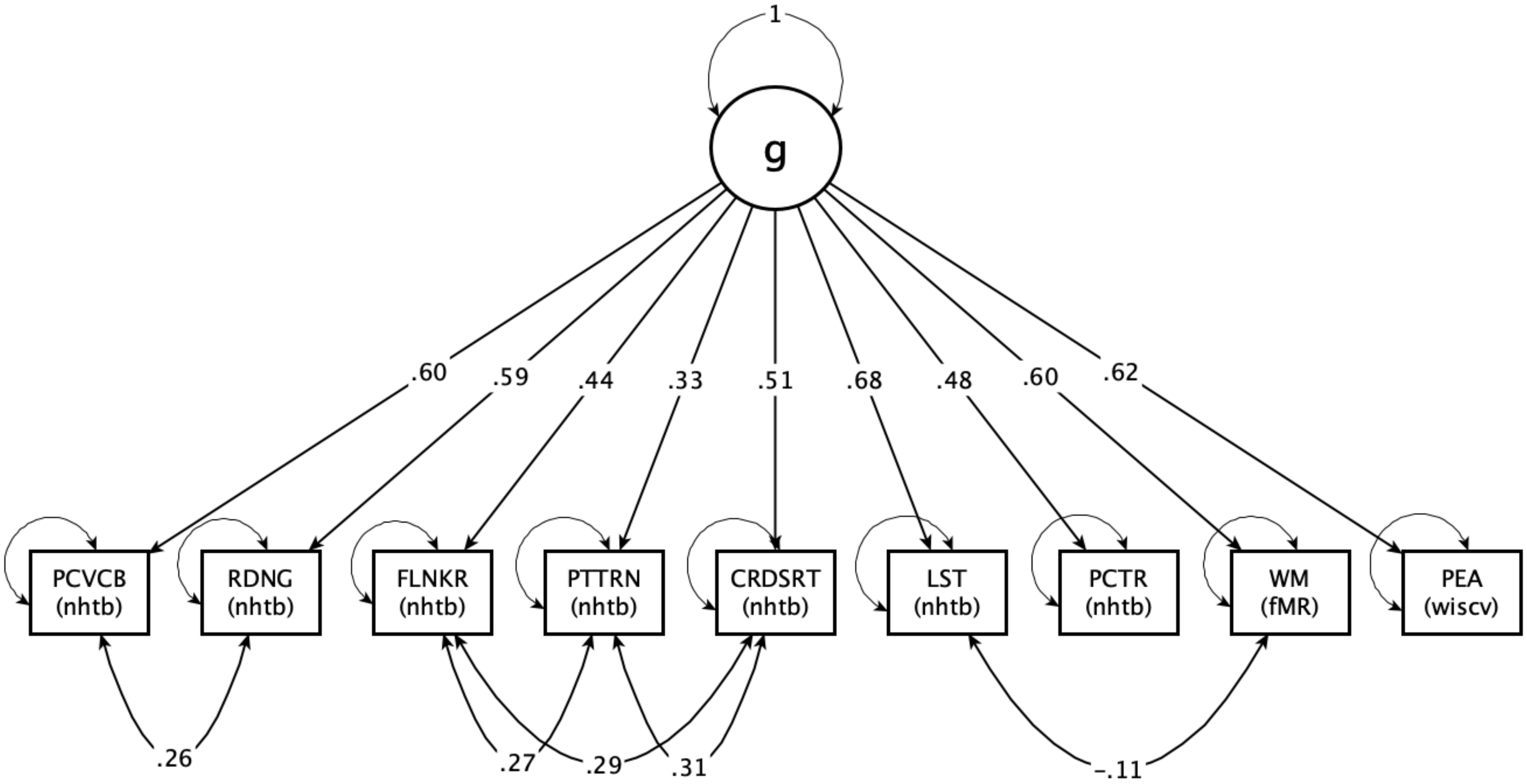


*Supplementary Figure 2: A single factor CFA was fit to estimate g with maximum likelihood estimation with robust (Huber-White) standard errors and FIML imputation (RMSEA = .032 & CFI = .987). Abbreviations are as follows; PCVCB = Picture vocabulary, RDNG = Oral reading recognition, FLNKR = Flanker task, PTTRN = Pattern comparison processing speed, CRDSRT = Card sorting task, LST = List sorting WM task, PCTR = Picture sequence memory test, WM = Overall nback accuracy, PEA = matrix reasoning total raw score.*

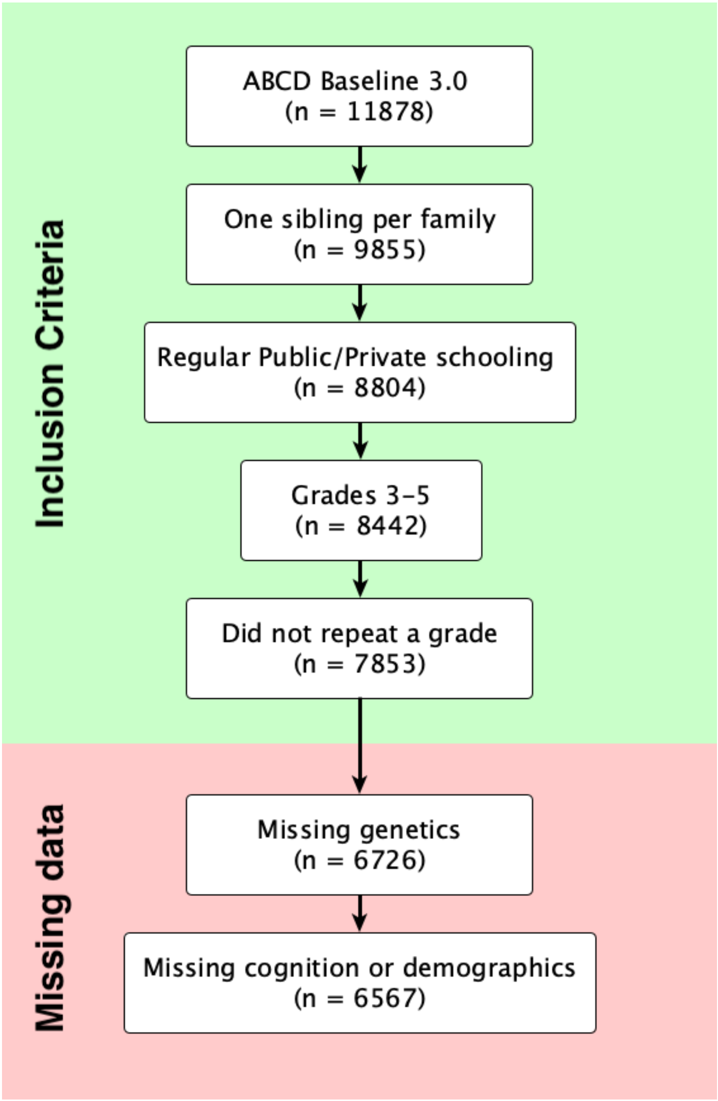


*Supplementary Figure 3: Sample selection flow chart, missing data is mainly genetic. Inclusion of those repeating grades bias the schooling coefficient upwards.*

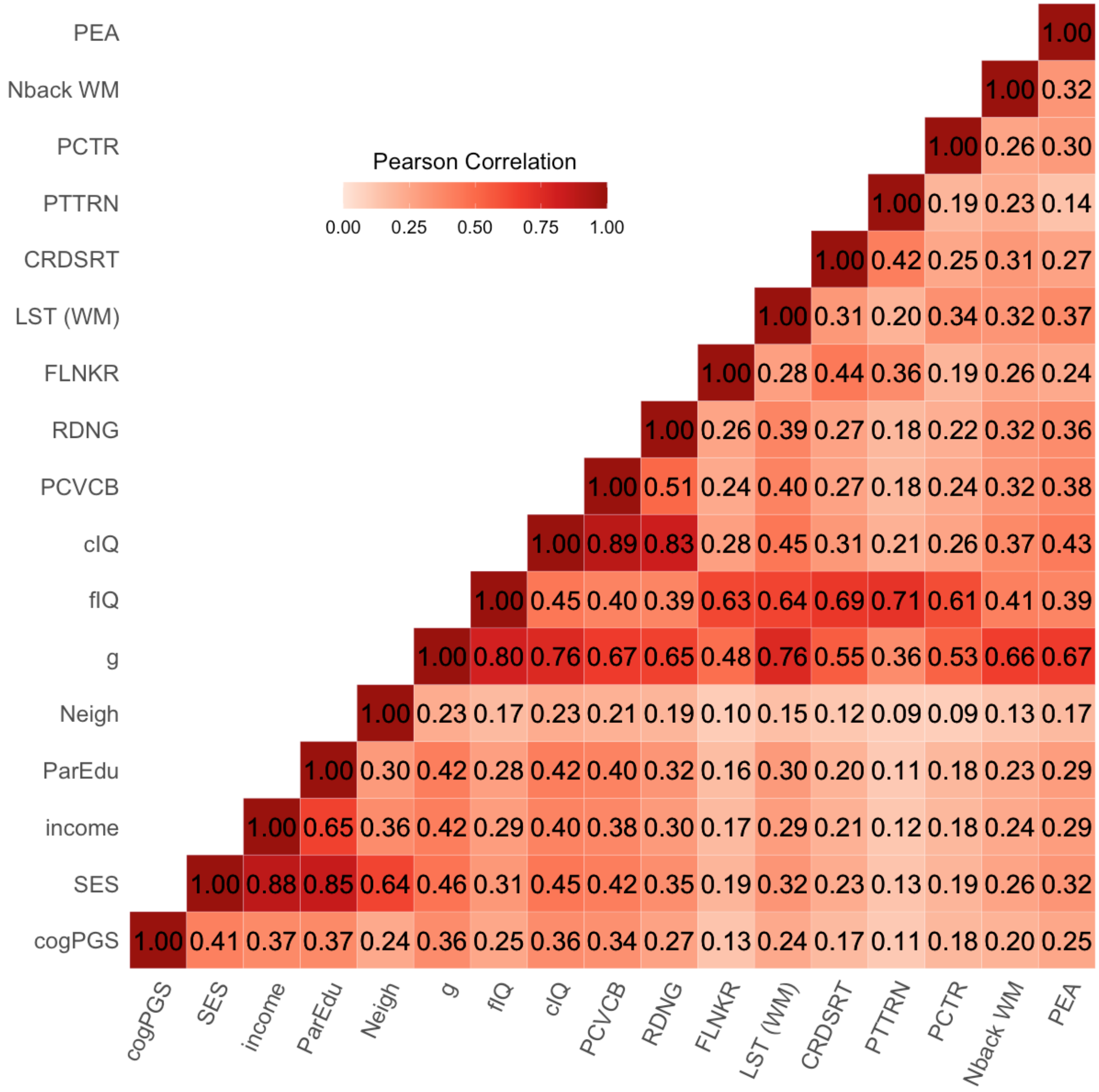


*Supplementary Figure 4* Correlation plot uncorrected for genetic PC's. All correlations are significant (<.0001) using Holm's multiple comparison correction.
