# Supplementary material for: Schooling substantially improves intelligence, but neither lessens nor widens the impacts of socioeconomics and genetics
Source: NPJ Sci Learn. 2022 Dec 15;7:33. doi: 10.1038/s41539-022-00148-5 (PMC9755250; doi:10.1038/s41539-022-00148-5)
Supplement: Supplementary file 2 — Reporting Summary [file 41539_2022_148_MOESM2_ESM.pdf]

nature portfolio

Corresponding author(s): Nicholas Judd & Torkel Klingberg

Last updated by author(s): Nov 5, 2021

Reporting Summary

Nature Portfolio wishes to improve the reproducibility of the work that we publish. This form provides structure for consistency and transparency in reporting. For further information on Nature Portfolio policies, see our Editorial Policies and the Editorial Policy Checklist.

Statistics

For all statistical analyses, confirm that the following items are present in the figure legend, table legend, main text, or Methods section.

n/a	Confirmed	
☐	☒	The exact sample size (*n*) for each experimental group/condition, given as a discrete number and unit of measurement
☐	☒	A statement on whether measurements were taken from distinct samples or whether the same sample was measured repeatedly
☐	☒	The statistical test(s) used AND whether they are one- or two-sided<br>*Only common tests should be described solely by name; describe more complex techniques in the Methods section.*
☐	☒	A description of all covariates tested
☐	☒	A description of any assumptions or corrections, such as tests of normality and adjustment for multiple comparisons
☐	☒	A full description of the statistical parameters including central tendency (e.g. means) or other basic estimates (e.g. regression coefficient) AND variation (e.g. standard deviation) or associated estimates of uncertainty (e.g. confidence intervals)
☐	☒	For null hypothesis testing, the test statistic (e.g. *F*, *t*, *r*) with confidence intervals, effect sizes, degrees of freedom and *P* value noted<br>*Give P values as exact values whenever suitable.*
☐	☒	For Bayesian analysis, information on the choice of priors and Markov chain Monte Carlo settings
☐	☒	For hierarchical and complex designs, identification of the appropriate level for tests and full reporting of outcomes
☐	☒	Estimates of effect sizes (e.g. Cohen's *d*, Pearson's *r*), indicating how they were calculated

Our web collection on statistics for biologists contains articles on many of the points above.

Software and code

Policy information about availability of computer code

Data collection	No software was used for data collection.
Data analysis	A reproducible R script (all analyses & figures) along with the necessary packages will be available at https://github.com/ABCDschooling upon publication.

For manuscripts utilizing custom algorithms or software that are central to the research but not yet described in published literature, software must be made available to editors and reviewers. We strongly encourage code deposition in a community repository (e.g. GitHub). See the Nature Portfolio guidelines for submitting code & software for further information.

Data

Policy information about availability of data

All manuscripts must include a data availability statement. This statement should provide the following information, where applicable:

- Accession codes, unique identifiers, or web links for publicly available datasets
- A description of any restrictions on data availability
- For clinical datasets or third party data, please ensure that the statement adheres to our policy

The ABCD study releases anonymized data annually to the research community accessed through the NIMH Data Archive (NDA), see https://abcdstudy.org/scientists_data_sharing.html for information on how to access it.

Field-specific reporting

Please select the one below that is the best fit for your research. If you are not sure, read the appropriate sections before making your selection.

☐ Life sciences ☒ Behavioural & social sciences ☐ Ecological, evolutionary & environmental sciences

For a reference copy of the document with all sections, see nature.com/documents/nr-reporting-summary-flat.pdf

Behavioural & social sciences study design

All studies must disclose on these points even when the disclosure is negative.

Study description	Quantitative data with a frequentest & Bayesian mixed effects regression discontinuity model.
Research sample	6,567 10-12 year old children representative of the United States for race, sex, SES and urbanicity.
Sampling strategy	Stratified sampling by the ABCD consortium, this is the largest cohort of its kind we included all children. Our Bayesian region of practical equivalence analysis discusses minimal effect sizes of interest, and shows our study is able to provide evidence for the null at an effect size of .05SDs. For more information see Garavan et al., 2018 for an overview of the ABCD design considerations and procedures.
Data collection	Data collection was completed by the ABCD consortium, the NIH behavioral tool box tasks used where collected with tablets (see Garavan et al., 2018 for more information). Income and parental education were self reported while neighborhood quality was based off census data from the address of primary residence.
Timing	See Garavan et al., 2018 for an overview of the ABCD design considerations and procedures.
Data exclusions	Exclusion criteria were pre-established. From the ABCD sample we excluded children that were not between grades 3-5 and enrolled in regular private or public schooling or repeated a grade. Those without genetic data were also excluded, we report how this changed the sample characteristics. To not bias our statistical analysis we had to randomly sample one sibling from families and excluded the other children from that family.
Non-participation	See Garavan et al., 2018 for an overview of the ABCD design considerations and procedures.
Randomization	None

Reporting for specific materials, systems and methods

We require information from authors about some types of materials, experimental systems and methods used in many studies. Here, indicate whether each material, system or method listed is relevant to your study. If you are not sure if a list item applies to your research, read the appropriate section before selecting a response.

Materials & experimental systems

n/a	Involved in the study	
☒	☐	Antibodies
☒	☐	Eukaryotic cell lines
☒	☐	Palaeontology and archaeology
☒	☐	Animals and other organisms
☐	☒	Human research participants
☒	☐	Clinical data
☒	☐	Dual use research of concern

Methods

n/a	Involved in the study	
☒	☐	ChIP-seq
☒	☐	Flow cytometry
☒	☐	MRI-based neuroimaging

Human research participants

Policy information about studies involving human research participants

Population characteristics	See above
Recruitment	Due to fundamental modeling limitations (i.e., a need to covary for ancestry based PCs) we had to exclude subjects missing genetic data. This biased our sample in respects to SES, we report this at the start of the results section.
Ethics oversight	UCSD

Note that full information on the approval of the study protocol must also be provided in the manuscript.
